# Lanostane Triterpenoids from Fruiting Bodies of *Ganoderma leucocontextum*

**DOI:** 10.1007/s13659-016-0089-3

**Published:** 2016-02-12

**Authors:** Zhen-Zhu Zhao, He-Ping Chen, Ying Huang, Zheng-Hui Li, Ling Zhang, Tao Feng, Ji-Kai Liu

**Affiliations:** State Key Laboratory of Phytochemistry and Plant Resources in West China, Kunming Institute of Botany, Chinese Academy of Sciences, Kunming, 650201 China; School of Pharmaceutical Sciences, South-Central University for Nationalities, Wuhan, 430074 China; University of Chinese Academy of Sciences, Beijing, 100049 China

**Keywords:** *Ganoderma leucocontextum*, Triterpenoids

## Abstract

**Abstract:**

Six new lanostane-type triterpenoids, namely leucocontextins S–X (1–6**)**, together with twelve known compounds, were isolated from the fruiting bodies of *Ganoderma leucocontextum*. Their structures were established by MS and NMR data.

**Graphical Abstract:**

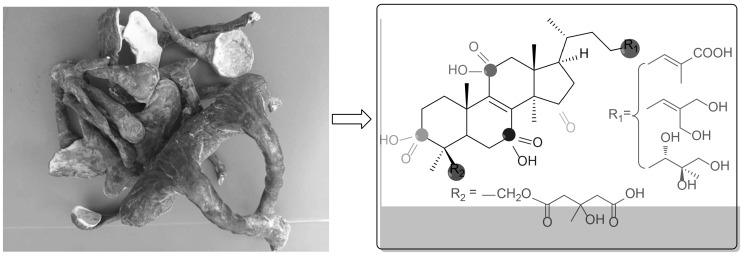

**Electronic supplementary material:**

The online version of this article (doi:10.1007/s13659-016-0089-3) contains supplementary material, which is available to authorized users.

## Introduction

**Fig. 1:**
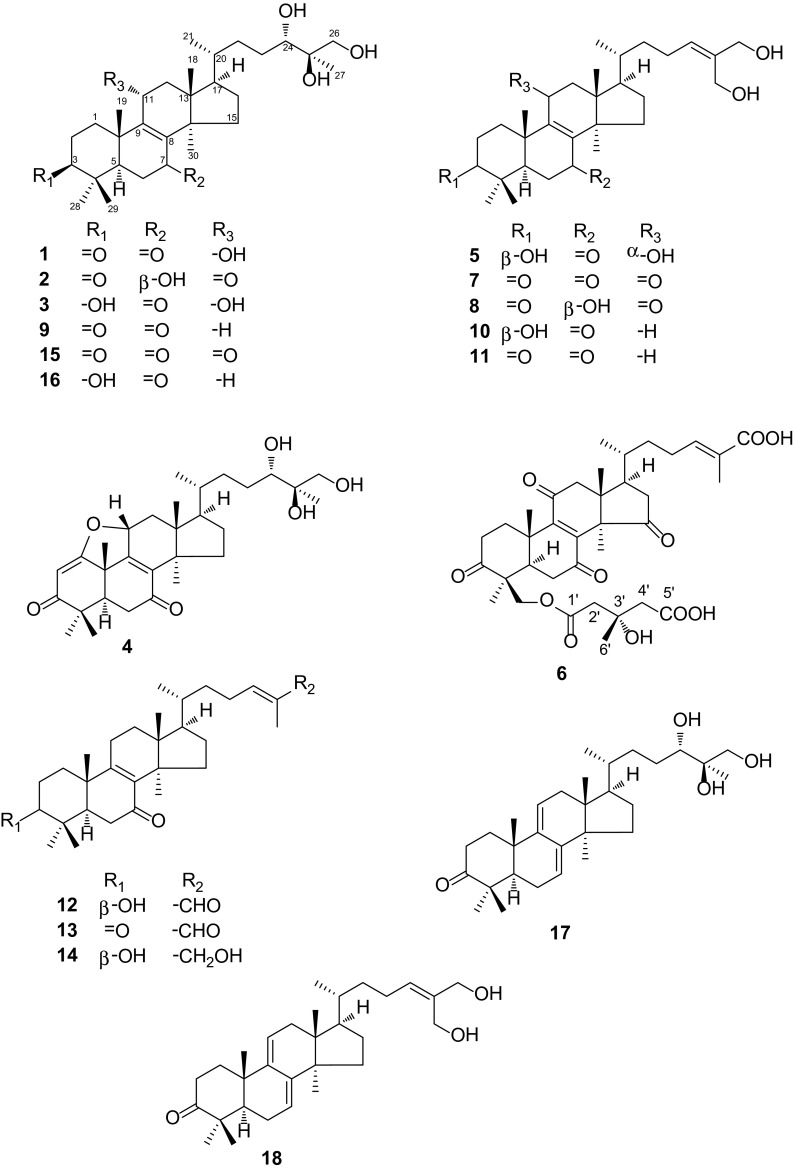
Structures of compounds 1–18

The higher fungi of *Ganoderma* are documented by many ancient Chinese Medicine books as tonic drugs for their health function. Modern pharmacological researches have illuminated that the effective components of the genus *Ganoderma* are mainly polysaccharides [1–3] and triterpenoids [4, 5]. Triterpenoids derived from *Ganoderma* spp., especially lanostane-type, have been one of the hotspots for a long time and the number of triterpenoids isolated from this genus has exceeded 300 [6–10]. *G. leucocontextum* is a special fungus because it is mainly distributed in plateau areas and has rarely been reported for its chemical compositions [6]. In the previous research, we have reported eighteen lanostane triterpenoids from this fungus [10]. In order to further make clear the secondary constituents of *G. leucocontextum* and comprehend its intrinsic differences from other common species, a further chemical investigation on the fruiting bodies of *G. leucocontextum* was carried out, which led to the discovery of six new lanostane triterpenoids, along with twelve known ones (Fig. 1). This study has shed light on the similarity of chemical structures between *G. leucocontextum* and other species, which are mostly lanostane type triterpenoids [11]. All the new compounds were evaluated for their cytotoxicities against human myelogenous leukemia (K562), hepatocellular carcinoma (SMMC-7721) and breast cancer (MCF-7) cells lines.

## Results and Discussion

**Fig. 2:**
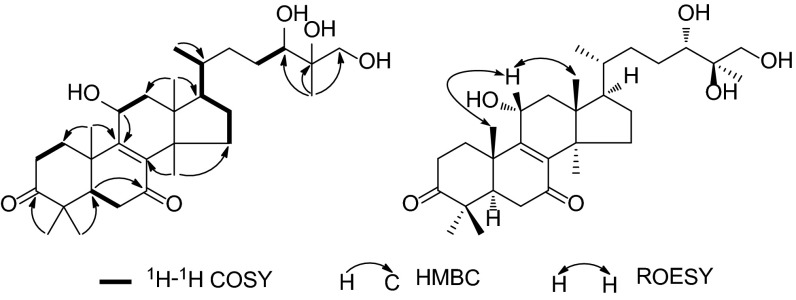
Key correlations in 2D NMR spectra of compound **1**

Leucocontextin S (**1**) was isolated as colorless needles. Its molecular formula was C_30_H_48_O_6_ established by HREIMS (*m/z* 504.3442 [M]^+^, calcd for 504.3451). The IR absorption bands at 3440, 3433, 1705 and 1640 cm^−1^ revealed the presence of hydroxy and a conjugated carbonyl group. Analyses of the ^1^H and ^13^C NMR data indicated the existence of seven methyls, nine methylenes (one oxygenated), a tetrasubstituted double bond, five methines (two oxygen-bonded), five *sp*^3^ quaternary carbons (one oxygenated), and two carbonyls (Tables 1, 2). The above-mentioned data suggested that compound **1** was quite similar to ganoderiol D (**9**) [12, 13], except for an extra hydroxy substituted at C-11 of **1**. This difference was confirmed by HMBC correlation of H-11 (*δ*_H_ 4.52)/C-9 (*δ*_C_ 159.1) and ^1^H-^1^H COSY correlation of H-11/H-12 (*δ*_H_ 1.87, 2.52) (Fig. 2). The orientation of 11-OH was established as *α* based on the correlations between H-11 and Me-18/Me-19 in the ROESY spectrum (Fig. 2). Furthermore, the absolute configurations of C-24 and C-25 was inferred as *S* and *R* via comparing with the chemical shifts of ganodermanontriol which had been synthesized in 2011 [14]. In the ^13^C NMR data (Table 2), signals at 79.3 (C-24), 74.2 (C-25) and 67.5 (C-26) were nearly identical with those of ganodermanontriol [79.3 (C-24), 74.0 (C-25), 67.6 (C-26)]. Thus, structure **1** was established as (24*S*,25*R*)-11*α*,24,25,26-tetrahydroxy-5*α*-lanost-8-ene-3,7-dione.

**Table 1:** ^1^H NMR spectral data of compounds **1**–**5** [*δ* in ppm, *J* in Hz]

No.	**1** ^*b*^	**2** ^*b*^	**3** ^*a*^	**4** ^*b*^	**5** ^*a*^
1	2.30, m	2.97, m	2.03, m		2.02, m
	2.19, m	1.72, m	1.90, m		1.89, m
2	2.72, m	2.62, m	1.72, m	5.28, s	1.72, m, 2H
	2.47, m	2.44, m	1.69, m		
3			3.20, m		3.24, t (7.6)
5	2.26, dd (14.5, 3.2)	2.10, dd (10.8, 4.9)	1.78, dd (14.7, 3.2)	2.12, dd (14.3, 3.2)	1.78, dd (14.5, 3.3)
6	2.60, dd (15.8, 14.5)	1.90, m	2.59, dd (14.7, 14.7)	2.53, dd (17.0, 14.3)	2.59, dd (15.7, 14.5)
	2.40, dd (15.8, 3.2)	1.69, m	2.40, dd (14.7, 3.2)	2.46, dd (17.0, 3.2)	2.39, dd (15.7, 3.3)
7		4.46, m			
11	4.52, dd (9.1, 5.2)		4.50, dd (9.2, 5.1)	5.36, dd (9.8, 7.7)	4.49, dd (9.3, 5.3)
12	2.52, dd (14.0, 9.1)	2.68, d (17.5)	2.43, dd (14.0, 9.2)	2.56, dd (12.1, 7.7)	2.43, dd (13.8, 9.3)
	1.87, dd (14.0, 5.2)	2.49, d (17.5)	1.89, dd (14.0, 3.2)	1.86, dd (12.1, 9.8)	1.89, dd (13.8, 5.3)
15	2.09, m	1.69, m	2.01, m	2.00, m	2.01, m
	1.61, m	1.20, m	1.61, m	1.74, m	1.60, m
16	2.00, m	2.10, m	2.01, m	2.07, m	1.98, m
	1.34, m	1.44, m	1.34, m	1.48, m	1.30, m
17	1.57, m	1.75, m	1.61, m	1.61, m	1.58, m
18	0.68, s	0.80, s	0.71, s	0.92, s	0.70, s
19	1.39, s	1.02, s	1.25, s	1.43, s	1.25, s
20	1.38, m	1.38, m	1.41, m	1.51, m	1.42, m
21	0.93, d (6.2)	0.93, d (6.4)	0.97, d (6.4)	1.03, d (6.3)	0.98, d (6.4)
22	1.83, m	1.84, m	1.83, m	1.86, m	1.54, m
	1.01, m	1.01, m	1.02, m	1.06, m	1.14, m
23	1.65, m	1.65, m	1.83, m	1.69, m	2.22, m
	1.22, m	1.22, m	1.16, m	1.25, m	2.05, m
24	3.44, d (10.3)	3.44, d (10.3)	3.38, d (10.3)	3.45, d (10.2)	5.56, t (7.3)
26	3.83, d (11.1)	3.83, d (11.1)	4.56, d (11.1)	3.84, d (11.1)	4.09, s, 2H
	3.47, d (11.1)	3.49, d (11.1)	3.45, d (11.1)	3.49, d (11.1)	
27	1.11, s	1.10, s	1.08, s	1.11, s	4.16, s, 2H
28	1.11, s	1.14, s	0.99, s	1.20, s	0.99, s
29	1.10, s	1.07, s	0.89, s	1.12, s	0.89, s
30	1.14, s	1.26, s	1.14, s	0.96, s	1.13, s

^*a*^ Data were measured at 600 MHz in CD_3_OD

^*b*^ Data were measured at 600 MHz in CDCl_3_

**Table 2:** ^13^C NMR spectral data of compounds **1**–**5** [*δ* in ppm, *J* in Hz]

No.	**1** ^*b*^	**2** ^*b*^	**3** ^*a*^	**4** ^*b*^	**5** ^*a*^
1	34.9, t	34.9, t	35.4, t	184.6, s	35.5, t
2	34.8, t	34.2, t	28.2, t	98.1, d	28.3, t
3	214.7, s	218.4, s	78.7, d	204.5, s	78.9, d
4	47.6, s	46.5, s	40.2, s	44.2, s	40.3, s
5	50.9, d	45.0, d	51.9, d	49.6, d	52.0, d
6	37.7, t	30.1, t	38.0, t	36.4, t	38.1, t
7	199.7, s	67.3, d	203.3, s	199.1, s	203.5, s
8	142.1, s	160.3,s	142.6, s	137.5, s	142.7, s
9	159.1, s	140.0,s	163.6, s	154.7, s	163.8, s
10	40.2, s	37.8, s	41.9, s	42.8, s	42.1, s
11	65.9, d	200.6, s	65.7, d	81.6, d	65.8, d
12	44.5, t	51.6, t	45.7, t	33.4, t	45.8, t
13	47.6, s	47.1, s	47.9, s	48.3, s	45.9, s
14	48.2, s	50.9, s	49.5, s	49.2, s	49.8, s
15	32.8, t	29.2,	34.0, t	29.6, t	34.2, t
16	28.1, t	27.3, t	29.0, t	28.3, t	29.2, t
17	50.0, d	50.4, d	51.3, d	48.5, d	51.4, d
18	17.1, q	17.1, q	17.5, q	16.9, q	17.6, q
19	19.4, q	17.8, q	20.3, q	18.4, q	20.5, q
20	36.8, d	36.8, d	38.0, d	36.6, d	37.5, d
21	18.9, q	18.9, q	19.3, q	19.4, q	19.1, q
22	33.5, t	33.5, t	34.6, t	33.7, t	37.5, t
23	28.9, t	29.1, t	28.5, t	29.0, t	25.3, t
24	79.3, d	79.4, d	77.0, d	79.3, d	131.1, d
25	74.2, s	74.0, s	75.5, s	74.0, s	139.1, s
26	67.5, t	67.8, t	68.9, t	67.8, t	65.7, t
27	21.2, q	21.2, q	18.7, q	21.2, q	58.4, t
28	21.7, q	27.6, q	28.0, q	21.2, q	28.2, q
29	25.1, q	20.6, q	15.9, q	29.0, q	16.0, q
30	25.4, q	27.8, q	25.3, q	26.0, q	25.3, q

^*a*^ Data were measured at 150 MHz in CD_3_OD

^*b*^ Data were measured at 150 MHz in CDCl_3_

 HREIMS data gave leucocontextin T (**2**) the same molecular formula as **1** (C_30_H_48_O_6_). Analysis of the 1D NMR data (Tables 1, 2) and HSQC displayed that **2** was an analogue of 11-oxo ganoderiol D (**15**) [7], while the carbonyl group at C-7 of **15** was reduced to a hydroxy in **2** (Supporting Information, Figure S12). And the orientation of 7-OH was *β* according to the ROESY correlation of H-7 (*δ*_H_ 4.46)/H-5 (*δ*_H_ 2.10) (Electronic supplementary material, Figure S13). Other chiral centers were same with those of **1** by comparison of the NMR data. Therefore, the structure of **2** was decided to be (24*S*,25*R*)-7*β*,24,25,26-tetrahydroxy-5*α*-lanost-8-ene-3,11-dione.

Leucocontextin U (**3**) was isolated as white powder. The HRESIMS of **3** showed an ion peak at *m/z* 507.3681 [M + H]^+^ (calcd for C_30_H_51_O_6_ 507.3680), suggesting a molecular formula C_30_H_50_O_6_. The NMR data of **3** suggested that it was a lanostane-type triterpenoid, which displayed similar characteristic signals to **1**. While the mainly difference between them was that the carbonyl located at C-3 in **1** was changed into a hydroxy group in **3**. This change was supported by HMBC correlations of Me-28 (*δ*_H_ 0.99)/C-3 (*δ*_C_ 78.7), Me-29 (*δ*_H_ 0.89)/C-3 (*δ*_C_ 78.7) and ^1^H-^1^H COSY correlations of H-2 (*δ*_H_ 1.72, 1.69)/H-3 (*δ*_H_ 3.20) (Electronic supplementary material, Figures S18, S19). Obvious cross peaks in the ROESY spectrum between H-3/H-5 (*δ*_H_ 1.78), H-11 (*δ*_H_ 4.50)/Me-18 (*δ*_H_ 0.71), and H-11/Me-19 (*δ*_H_ 1.25) suggested that the orientations of C-3 and C-11 were *β* and *α*, respectively. The stereochemistry of other chiral centers of **3** was same with those of **1**. Hence, structure of **3** was elucidated to be (24*S*,25*R*)-3*β,*11*α*,24,25,26-pentahydroxy-5*α*-lanost-8-en-7-one.

Leucocontextin V (**4**) had the molecular formula of C_30_H_44_O_6_ as determined by HRESIMS based on the ion peak at *m/z* 501.3207 [M + H]^+^ (calcd for 501.3211), requiring nine degrees of unsaturation. The 1D NMR data of **4** showed similarities to those of **1**. In compound **4**, the HMBC correlations from H-2 (*δ*_H_ 5.28, s) to C-1 (*δ*_C_ 184.6), C-4 (*δ*_C_ 44.2) and C-10 (*δ*_C_ 42.8) suggested that an *α*,*β*-unsaturated ketone group was located at ring A and C-1 was oxygenated (Electronic supplementary material, Figure S26). Besides, the chemical shift of C-11 (*δ*_C_ 81.6) of **4** was downfield shifted comparing to that of **1** (*δ*_C_ 65.9). Therefore, an ether linkage was assigned between C-1 and C-11 based on aforementioned evidences and the degrees of unsaturation [15, 16]. ROESY experiment showed correlations between H-11 (*δ*_H_ 5.36)/Me-18 (*δ*_H_ 0.92) and H-11/Me-19 (*δ*_H_ 1.43), which revealed that the orientation of C-11 was *α*. The relative configuration of **4** was same with **1** (Supporting Information, Figure S27). Consequently, the structure of **4** was determined to be (24*S*,25*R*)-1,11*α*-epoxy-24,25,26-trihydroxy-5*α*-lanosta-1,8-diene-3,7-dione.

Leucocontextin W (**5**) possessed the molecular formula of C_30_H_48_O_5_ according to the HRESIMS analysis at *m/z* 489.3575 [M + H]^+^ (calcd for C_30_H_49_O_5_, 489.3575). Analysis of its 1D NMR spectra showed that the structure of compound **5** was similar to ganoleucoin G (**7**) [6] (Tables 1, 2), with the differences being that the carbonyl groups at C-3 and C-11 were reduced to hydroxy groups in **5**. These changes were supported by the HMBC correlations from Me-28 (*δ*_H_ 0.99) and Me-29 (*δ*_H_ 0.89) to C-3 (*δ*_C_ 78.9) and correlations from H-12 (*δ*_H_ 2.43; 1.89) to C-11 (*δ*_C_ 65.8) (Electronic supplementary material, Figures S32, S33), as well as the HRESIMS report, which showed the molecular weight of **5** was 4 amu more than that of **7**. The orientations of 3-OH and 11-OH were established as *β* and *α*, respectively, based on ROESY correlations of H-3 (*δ*_H_ 3.24)/Me-28 (*δ*_H_ 0.99) and H-11 (*δ*_H_ 4.49)/Me-19 (*δ*_H_ 1.25) (Supporting Information, Figure S34). Thus the structure was defined as 3*β*,11*α*,26,27-tetrahydroxy-5*α*-lanosta-8,24-dien-7-one.

Leucocontextin X (**6**) gave a molecular ion peak at *m/z* 656.3188 [M]^+^ in its HREIMS spectrum, indicating a molecular formula of C_36_H_48_O_11_ (13 degrees of unsaturation). The 1D NMR (Table 3) displayed seven methyls (*δ*_H_ 0.86, 1.03, 1.22 1.25, 1.36, 1.43, 1.63; *δ*_C_ 16.5, 18.8, 22.2, 12.5, 27.8, 18.9, 21.3), ten *sp*^3^ methylenes (one oxygenated, *δ*_C_ 66.6), three *sp*^3^ methines, five *sp*^3^ quaternary carbons (one oxygenated, *δ*_C_ 70.8), a tetrasubstituted and a trisubstituted double bonds as well as seven carbonyls (*δ*_C_ 171.7, 172.3, 175.9, 200.7, 201.2, 210.5 and 213.9). Furthermore, the aforementioned data, together with HMBC and ^1^H-^1^H COSY spectral signals, showed that structure of **6** was closely related to ganoleucion L [6], difference being that the (4-carboxy-3-hydroxy-3-methylbutanoyl)oxy group was substituted at C-29 based on HMBC correlations from H-29 (*δ*_H_ 4.61 and 4.62) to C-1′ (*δ*_C_ 172.3) (Supporting Information, Figure S39). The absolute configuration of C-3′ was inferred as *S* by comparing the carbon chemical shifts of (4-carboxy-3-hydroxy-3-methylbutanoyl)oxy group between **6** and those of ganoleucion L in the same NMR solvent (Supporting Information, Figure S43). As a result, the structure of **6** was elucidated as (24*E*)-29-*O*-((3*S*)-4-carboxy-3-hydroxy-3-methylbutanoyl)-3,7,11,15-tetraoxo-5*α*-lanosta-8,24-dien-26-oic acid.

**Table 3:** ^1^H and ^13^C NMR spectral data of compound 6 [*δ* in ppm, *J* in Hz]

No.	*δ*_C_	*δ*_H_	*No.*	*δ*_C_	*δ*_H_
1	35.4, t	2.96, m	19	18.9, q	1.43, s
		1.77, m			
2	35.6, t	2.66, m	20	36.8, d	1.58, m
		2.61, m			
3	213.9, s		21	18.8, q	1.03, d (6.9)
4	51.9, s		22	35.4, t	1.55, m
					1.28, m
5	52.6, d	2.42, dd (15.2, 2.9)	23	26.4, t	2.31, m
					2.19, m
6	38.0, t	2.87, m	24	143.8, d	6.77, t (7.0)
		2.61, m			
7	200.7, s		25	129.1, s	
8	147.9, s		26	171.7, s	
9	151.5, s		27	12.5, q	1.82, s
10	40.7, s		28	22.2, q	1.22, s
11	201.2, s		29	66.6, t	4.61, d (12.2)
					4.14, d (12.2)
12	50.1, t	3.08, d (15.9)	30	21.3, q	1.63, s
		2.73, d (15.9)			
13	45.3, s		1′	172.3, s	
14	58.5, s		2′	46.2, t	2.67, s, 2H
15	210.5, s		3′	70.8, s	
16	41.1, t	2.89, m	4′	46.2, t	2.61, s, 2H
		1.89, dd (18.3, 8.1)			
17	46.1, d	2.27, m	5′	175.9, s	
18	16.5, q	0.86, s	6′	27.8, q	1.36, s

*δ*_H_ Data were measured at 600 MHz in CD_3_OD

*δ*_C_ Data were measured at 150 MHz in CD_3_OD

Twelve known lanostane-type triterpeniods, ganoleucoins G (**7**) [6] and I (**8**) [6], ganoderiols D (**9**) [12], E (**10**) [12], F (**18**) [12], H (**16**) [12] and J (**11**) [13], lucialdehyde B (**13**) [17], lucidadiol (**14**) [18], lucidal (**12**) [18], 11-oxo ganoderiol D (**15**) [7] and ganodermanontriol (**17)** [12, 14] were also obtained from this fungus. Their structures were identified by 1D NMR spectrum as well as comparison with reported data.

Compounds **1-6** were evaluated for inhibitory activities against K562, SMMC-7721 and MCF-7 cell lines. The cytotoxicity was determined by using MTS method. Unfortunately, none of them showed significant activity.

## Experimental Section

### General Experimental Procedures

Optical rotations were obtained on a JASCO P-1020 digital polarimeter (Horiba, Kyoto, Japan). UV spectra were recorded on a Shimadzu UV-2401PC (Shimadzu, Kyoto, Japan). 1D and 2D NMR spectra were obtained on a Bruker Avance III 600 MHz spectrometer (Bruker Biospin GmbH, Karlsruhe, Germany). HREIMS was measured on Waters Xevo TQ-S and Waters Autospec Premier P776 mass spectrometers (Waters, Milford, MA, USA). HRESIMS were recorded on an Agilent 6200 Q-TOF MS system (Agilent Technologies, Santa Clara, CA, USA). Melting points were measured on an X-4 microscopic melting point meter (Yuhua Instrument Co., Ltd, Gongyi, China). Sephadex LH-20 (Amersham Biosciences, Upssala, Sweden) and silica gel (Qingdao Haiyang Chemical Co., Ltd) were used for column chromatography (CC). Medium Pressure Liquid Chromatography (MPLC) was performed on a Büchi Sepacore System equipping with pump manager C-615, pump modules C-605 and fraction collector C-660 (Büchi Labortechnik AG, Flawil, Switzerland), and columns packed with Chromatorex C-18 (40–75 μm, Fuji Silysia Chemical Ltd., Kasugai, Japan). Preparative High Performance Liquid Chromatography (prep-HPLC) was performed on an Agilent 1260 liquid chromatography system equipped with Zorbax SB-C18 columns (5 μm, 9.4 mm × 150 mm or 21.2 mm × 150 mm) and a DAD detector (Agilent Technologies, Santa Clara, CA, USA).

### Fungal Material

The fungus *G. leucocontextum* were collected in Nyingchi, Tibet, China in 2014, and identified by professor Yu-Cheng Dai (Beijing Forestry University). A voucher specimen of *G. leucocontextum* was deposited in the Herbarium of Kunming Institute of Botany, Chinese Academy of Sciences (No. HFC 20140613).

### Extraction and Isolation

The air-dried and powdered fruiting bodies of *G. leucocontextum* (2.5 kg) was macerated three times with 95 % methanol. The extract was evaporated under reduced pressure and partitioned between EtOAc and water four times to give a crude extract (65 g). The crude extract was subject to MPLC with a stepwise gradient of MeOH/H_2_O (v/v 40:60–100:0) to afford nine fractions (A–I).

Fraction D (10.5 g) was separated by Sephadex LH-20 (MeOH) to give two major subfractions (D1-D2). Subfraction D1 was separated on silica gel CC using a petroleum ether-acetone gradient solvent system (v/v, 4:1–2:1) to obtain three subfractions (D1a-D1c). Subfraction D1a and D1b were separated by Sephadex LH-20 (acetone) to afford three subfractions (D1a1–D1a3 and D1b1–D1b3), respectively. Each part was purified on prep-HPLC (MeCN-H_2_O 20–45 %, 25 min) to yield compounds **10** (8.0 mg), **11** (9.4 mg), **12** (7.3 mg), **13** (11.5 mg), **14** (21.0 mg), and **18** (15.4 mg). Fraction E (12.5 g) was separated on Sephadex LH-20 (MeOH) to give four subfractions (E1–E4). E1 was subjected to silica gel CC with a petroleum ether-acetone gradient solvent system (v/v, 4:1–2:1) to obtain six subfractions (E1a–E1f). Compounds **7** (1.5 mg) and **8** (3.8 mg) were isolated from E1e by prep-HPLC (MeCN-H_2_O 24–39 %, 30 min). E1f was subjected on prep-HPLC (MeCN–H_2_O 21–41 %, 25 min) to afford **6** (19.7 mg), **1** (5.3 mg), **2** (4.3 mg).

Fraction F (11.3 g) was separated on Sephadex LH-20 (MeOH) to give three sections (F1–F3) and F1 was separated by MPLC with MeOH–H_2_O (v/v 40:60–80:20) to give four subfractions (F1a–F1d). Then fraction F1b was subjected to Sephadex LH-20 (acetone) to give three subfractions (F1b1–F1b3) and each of them was purified by prep-HPLC with the mobile phase MeCN/H_2_O (25–45 %, 25 min) to afford compounds **3** (2.0 mg), **4** (2.9 mg), **5** (3.1 mg), **9** (24.5 mg), **15** (8.9 mg), **16** (13.2 mg) and **17** (11.0 mg).

### Leucocontextin S (1)

Colorless needles; m.p. 208.2-209.4; [α]_D_^17^ −18.13 (*c* 0.40, MeOH). UV (MeOH) λ_max_ nm (log *ε*): 250.8 (3.82). IR (KBr) ν_max_ cm^−1^: 3440, 3433, 2955, 2924, 2853, 1705, 1640, 1461, 1383, 1112, 1036; ^1^H NMR (600 MHz, CDCl_3_) and ^13^C NMR (150 MHz, CDCl_3_) data, Tables 1, 2, HREIMS *m/z*: 504.3442 [M]^+^ (calcd for C_30_H_48_O_6_, 504.3451).

### Leucocontextin T (2)

White powder; [α]_D_^22^ +141.22 (*c* 0.30, MeOH). UV (MeOH) λ_max_ nm (log *ε*): 254.0 (3.75). IR (KBr) ν_max_ cm^−1^: 3424, 2969, 2938, 2884, 1701, 1657, 1636, 1463, 1384, 1040; ^1^H NMR (600 MHz, CDCl_3_) and ^13^C NMR (150 MHz, CDCl_3_) data, Tables 1 and 2, HREIMS *m/z*: 504.3459 [M]^+^ (calcd for C_30_H_48_O_6_, 504.3451).

### Leucocontextin U (3)

White powder; [α]_D_^17^ −29.44 (*c* 0.06, MeOH). UV (MeOH) λ_max_ nm (log *ε*): 252.4 (3.56). IR (KBr) ν_max_ cm^−1^: 3446, 2960, 2923, 1647, 1461, 1384, 1035; ^1^H NMR (600 MHz, CD_3_OD) and ^13^C NMR (150 MHz, CD_3_OD) data, Tables 1 and 2, HRESIMS *m/z*: 507.3681 [M + H]^+^ (calcd for C_30_H_51_O_6_, 507.3680).

### Leucocontextin V (4)

White powder; [α]_D_^24^ −73.83 (*c* 0.20, MeOH). UV (MeOH) λ_max_ nm (log *ε*): 257.0 (4.02), 382.0 (2.89). IR (KBr) ν_max_ cm^−1^: 3440, 2962, 2926, 1632, 1461, 1384, 1042; ^1^H NMR (600 MHz, CDCl_3_) and ^13^C NMR (150 MHz, CDCl_3_) data, Tables 1 and 2, HRESIMS *m/z*: 501.3207 [M + H]^+^(calcd for C_30_H_45_O_6_, 501.3211).

### Leucocontextin W (5)

Colorless needles; m.p. 179.3–186.4; [α]_D_^23^ +1.75 (*c* 0.01, MeOH). UV (MeOH) λ_max_ nm (log *ε*): 252.0 (3.80). IR (KBr) ν_max_ cm^−1^: 3446, 2955, 2924, 2853, 1641, 1461, 1383, 1112, 1036; ^1^H NMR (600 MHz, CD_3_OD) and ^13^C NMR (150 MHz, CD_3_OD) data, Tables 1, 2, HRESIMS *m/z*: 489.3575 [M + H]^+^ (calcd for C_30_H_49_O_5_, 489.3575).

### Leucocontextin X (6)

Yellow powder; [α]_D_^21^ +111.92 (*c* 0.26, MeOH). UV (MeOH) λ_max_ nm (log *ε*): 213.6 (4.16), 253.4 (3.80). IR (KBr) ν_max_ cm^−1^: 3433, 3086, 2976, 2936, 1746, 1701, 1684, 1462, 1414, 1385, 1229, 1184, 1014. ^1^H NMR (600 MHz, CD_3_OD) and ^13^C NMR (150 MHz, CD_3_OD) data, Table 3, HREIMS *m/z*: 656.3188 [M]^+^ (calcd for C_36_H_48_O_11_, 656.3197).

### Cytotoxicity Assay

The cytotoxicity against K562, SMMC-7721 and MCF-7 cells lines of compounds **1-6** were tested by using MTS method. MTS [3-(4,5-dimethylthiazol-2-yl)-5(3-carboxymethoxyphenyl)-2-(4-sulfopheny)-2*H*-tetrazolium]is an analogue of MTT [19], which can be reduced into soluble formazan by succinate dehydrogenase in mitochondria of living cells. Moreover, the optical density value of formazan (490 nm) is proportional to the number of living cells (Electronic supplementary material, Table S1).

## Electronic supplementary material

Supplementary material 1 (PDF 6053 kb)

## References

[1] Zhang S, Nie S, Huang D, Huang J, Feng Y, Xie M (2014). J. Agric. Food Chem..

[2] Pillai TG, Uma Devi P (2013). Mutat. Res. Genet. Toxicol. Environ. Mutagen..

[3] Saravanakumar P, Karthikeyan V, Patharajan S, Kannan T, Sathya K, Kalaichelvan PT (2010). Pharmacologyonline.

[4] Rios JL, Andujar I, Recio MC, Giner RM (2012). J. Nat. Prod..

[5] Yeh SF, Lee KC, Shiao MS (1987). Proc. Natl. Sci. Counc. Repub. China Part A.

[6] Wang K, Bao L, Ma WK, Xiong J, Han W, Wang W, Yin H, Liu J (2015). Nat. Prod..

[7] Zhang SS, Ma QY, Huang SZ, Dai HF, Guo ZK, Yu ZF, Zhao YX (2015). Phytochemistry.

[8] Zhao ZZ, Yin RH, Chen HP, Feng T, Li ZH, Dong ZJ, Cui BK, Liu JK (2015). J. Asian Nat. Prod. Res..

[9] Peng X, Liu J, Xia J, Wang C, Li X, Deng Y, Bao N, Zhang Z, Qiu MH (2015). Phytochemistry.

[10] Zhao ZZ, Chen HP, Li ZH, Dong ZJ, Bai X, Zhou ZY, Feng T, Liu JK (2016). Fitoterapia.

[11] Xia Q, Zhang H, Sun X, Zhao H, Wu L, Zhu D, Yang G, Shao Y, Zhang X, Mao X, Zhang L (2014). Molecules.

[12] Nishitoba T, Oda K, Sato H, Sakamura S (1988). Agric. Biol. Chem..

[13] Liu JQ, Wang CF, Li Y, Luo HR, Qiu MH (2012). Planta Med..

[14] Kennedy EM, P’Pool SJ, Jiang J, Sliva D, Minto RE (2011). J. Nat. Prod..

[15] Banskota AH, Tezuka Y, Tran KQ, Tanaka K, Saiki I, Kadota S (2000). Chem. Pharm. Bull..

[16] Silverstein RM, Webster FX, Kiemle DJ (2005). Spectrometric identification of organic compounds.

[17] Gao JJ, Min BS, Ahn EM, Nakamura N, Lee HK, Hattori M (2002). Chem. Pharm. Bull..

[18] González AG, León F, Rivera A, Muñoz CM, Bermejo J (1999). J. Nat. Prod..

[19] Mosmann T (1983). J. Immunol. Methods.

