# Supplementary material for: Lanostane Triterpenoids from Fruiting Bodies of Ganoderma leucocontextum
Source: Nat Prod Bioprospect. 2016 Feb 12;6(2):103–9. doi: 10.1007/s13659-016-0089-3 (PMC4805654; doi:10.1007/s13659-016-0089-3)
Supplement: Supplementary file 1 — Supplementary material 1 (PDF 6053 kb) [file 13659_2016_89_MOESM1_ESM.pdf]

Supporting information

Lanostane Triterpenoids from Fruiting Bodies of *Ganoderma leucocontextum*

Zhen-Zhu Zhao[a,c], He-Ping Chen[a,c], Ying Huang[a,c], Zheng-Hui Li[b], Ling Zhang[a], Tao Feng[b,]* and Ji-Kai Liu[a,b,]*

[a] *State Key Laboratory of Phytochemistry and Plant Resources in West China, Kunming Institute of Botany, Chinese Academy of Sciences, Kunming 650201, China*

[b] *School of Pharmaceutical Sciences, South-Central University for Nationalities, Wuhan 430074, China*

[c] *University of Chinese Academy of Sciences, Beijing 100049, China*

*Corresponding author. Kunming Institute of Botany, Chinese Academy of Sciences, Kunming 650201, P. R. China Tel.: +86 871 5216327; fax: +86 871 5150227.

E-mail addresses: tfeng@mail.scuec.edu.cn (T. Feng); jkliu@mail.kib.ac.cn (J.K. Liu).

Figures	Content
S1	^{1}H NMR spectrum of compound **1** ($CDCl_3$).
S2	^{13}C NMR and DEPT spectra of compound **1** ($CDCl_3$).
S3	HSQC spectrum of compound **1** ($CDCl_3$).
S4	^{1}H-^{1}H COSY spectrum of compound **1** ($CDCl_3$)
S5	HMBC spectrum of compound **1** ($CDCl_3$).
S6	ROESY spectrum of compound **1** ($CDCl_3$).
S7	HREIMS spectrum of compound **1**
S8	^{1}H NMR spectrum of compound **2** ($CDCl_3$).
S9	^{13}C NMR and DEPT spectra of compound **2** ($CDCl_3$).
S10	HSQC spectrum of compound **2** ($CDCl_3$).
S11	^{1}H-^{1}H COSY spectrum of compound **2** ($CDCl_3$)
S12	HMBC spectrum of compound **2** ($CDCl_3$).
S13	ROESY spectrum of compound **2** ($CDCl_3$).
S14	HREIMS spectrum of compound **2**
S15	^{1}H NMR spectrum of compound **3** (CD_3OD).
S16	^{13}C NMR and DEPT spectra of compound **3**(CD_3OD).
S17	HSQC spectrum of compound **3** (CD_3OD).
S18	^{1}H-^{1}H COSY spectrum of compound **3** (CD_3OD)
S19	HMBC spectrum of compound **3** (CD_3OD).
S20	ROESY spectrum of compound **3** (CD_3OD).
S21	HRESIMS spectrum of compound **3**
S22	^{1}H NMR spectrum of compound **4** ($CDCl_3$).
S23	^{13}C NMR and DEPT spectra of compound **4** ($CDCl_3$).
S24	HSQC spectrum of compound **4** ($CDCl_3$).
S25	^{1}H-^{1}H COSY spectrum of compound **4** ($CDCl_3$)
S26	HMBC spectrum of compound **4** ($CDCl_3$).
S27	ROESY spectrum of compound **4** ($CDCl_3$).
S28	HRESIMS spectrum of compound **4**
S29	^{1}H NMR spectrum of compound **5** (CD_3OD).
S30	^{13}C NMR and DEPT spectra of compound **5** (CD_3OD).
S31	HSQC spectrum of compound **5** (CD_3OD).
S32	^{1}H-^{1}H COSY spectrum of compound **5** (CD_3OD)
S33	HMBC spectrum of compound **5** (CD_3OD).
S34	ROESY spectrum of compound **5** (CD_3OD).
S35	HRESIMS spectrum of compound **5**
S36	^{1}H NMR spectrum of compound **6** (CD_3OD).
S37	^{13}C NMR and DEPT spectra of compound **6** (CD_3OD).

S38	HSQC spectrum of compound **6** (CD_3OD).
S39	^{1}H-^{1}H COSY spectrum of compound **6** (CD_3OD)
S40	HMBC spectrum of compound **6** (CD_3OD).
S41	ROESY spectrum of compound **6** (CD_3OD).
S42	HREIMS spectrum of compound **6.**
S43	^{13}C NMR spectra of **6** and ganoleucoin L.

S1 Bioassy

S1.1 Cytotoxicity Assay

The cytotoxicity assay was performed according to the MTS method in 96-well microplates. Three human cancer cell lines: human myelogenous leukemia (K562), hepatocellular carcinoma (SMMC-7721) and breast cancer (MCF-7) cells were used in the cytotoxicity assay. All the cells were cultured in RPMI-1640 or DMEM (Hyclone, Logan, UT, USA), supplemented with 10% fetal bovine serum (Hyclone) in 5% CO_2 at 37 °C. Briefly, 100 μL of adherent cells were seeded into each well of 96-well cell culture plates and allowed to adhere for 12 h before drug addition, while suspended cells were seeded just before drug addiction with an initial density of 1 × 105 cells/mL. Each tumor cell line was exposed to the test compound at concentrations of 0.064, 0.32, 1.6, 8, and 40 μmol in triplicates for 48 h, with cisplatin (sigma, USA) as a positive control After compound treatment, cell viability was detected and cell growth curve was graphed. In these tests, all the experiments were performed in triplicate, and the results were expressed in IC_{50} as calculated by the Logit method.

Table S1 Cytotoxicity of compounds **1-6** against K562, SMMC-7721 and MCF-7 cell lines (IC_{50} μM).

**Sample**	**K562**	**SMMC-7721**	**MCF-7**
**1**	>40	>40	>40
**2**	>40	>40	>40
**3**	>40	>40	>40
**4**	>40	>40	>40
**5**	>40	>40	>40
**6**	>40	>40	>40
**DDP(MW300)**	14.73	14.28	15.31
**Taxol**	<0.008	<0.008	<0.008

Figure S1. ^{1}H NMR spectrum of compound **1** ($CDCl_3$).

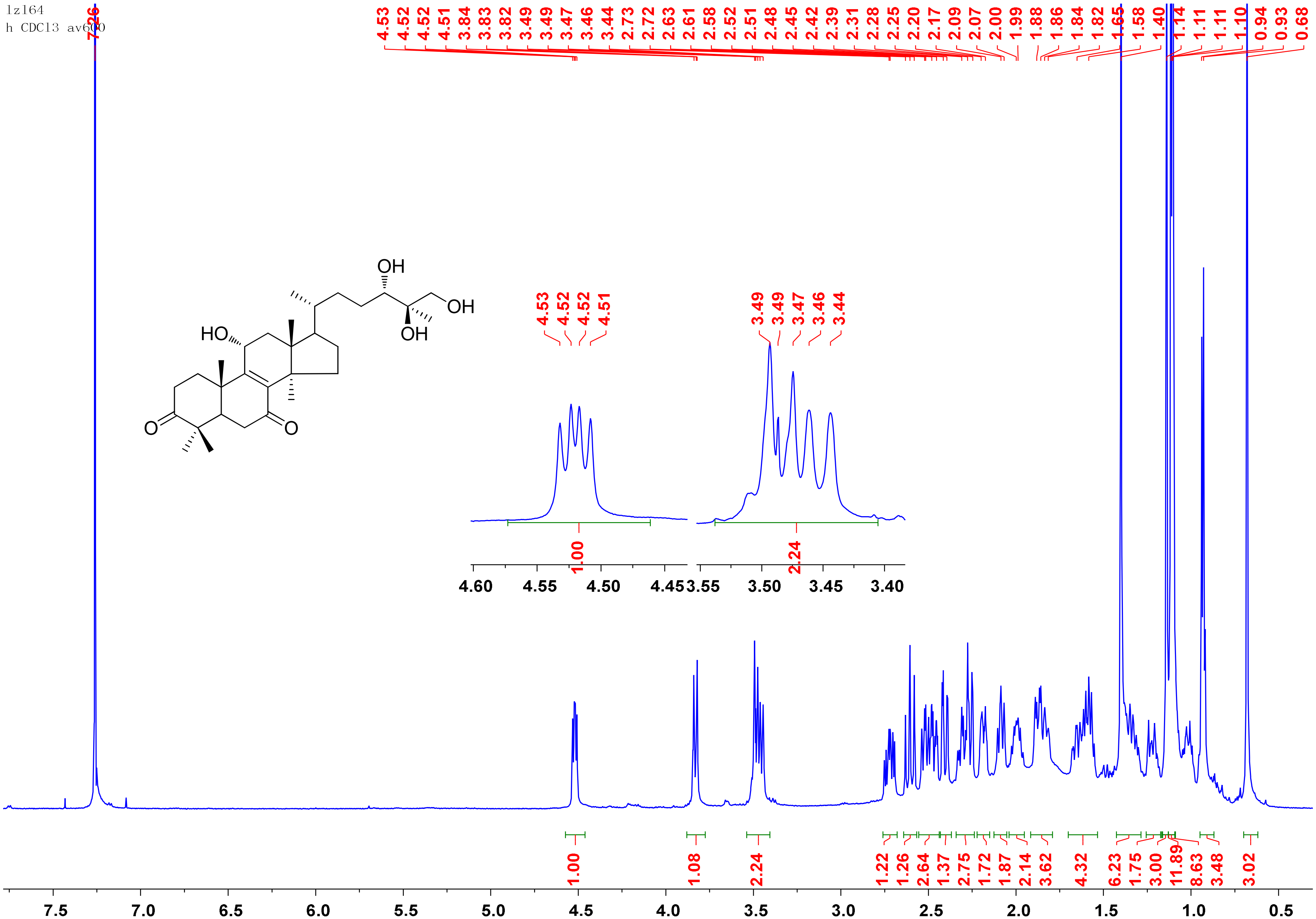

Figure S2. ^{13}C NMR and DEPT spectra of compound **1** ($CDCl_3$).

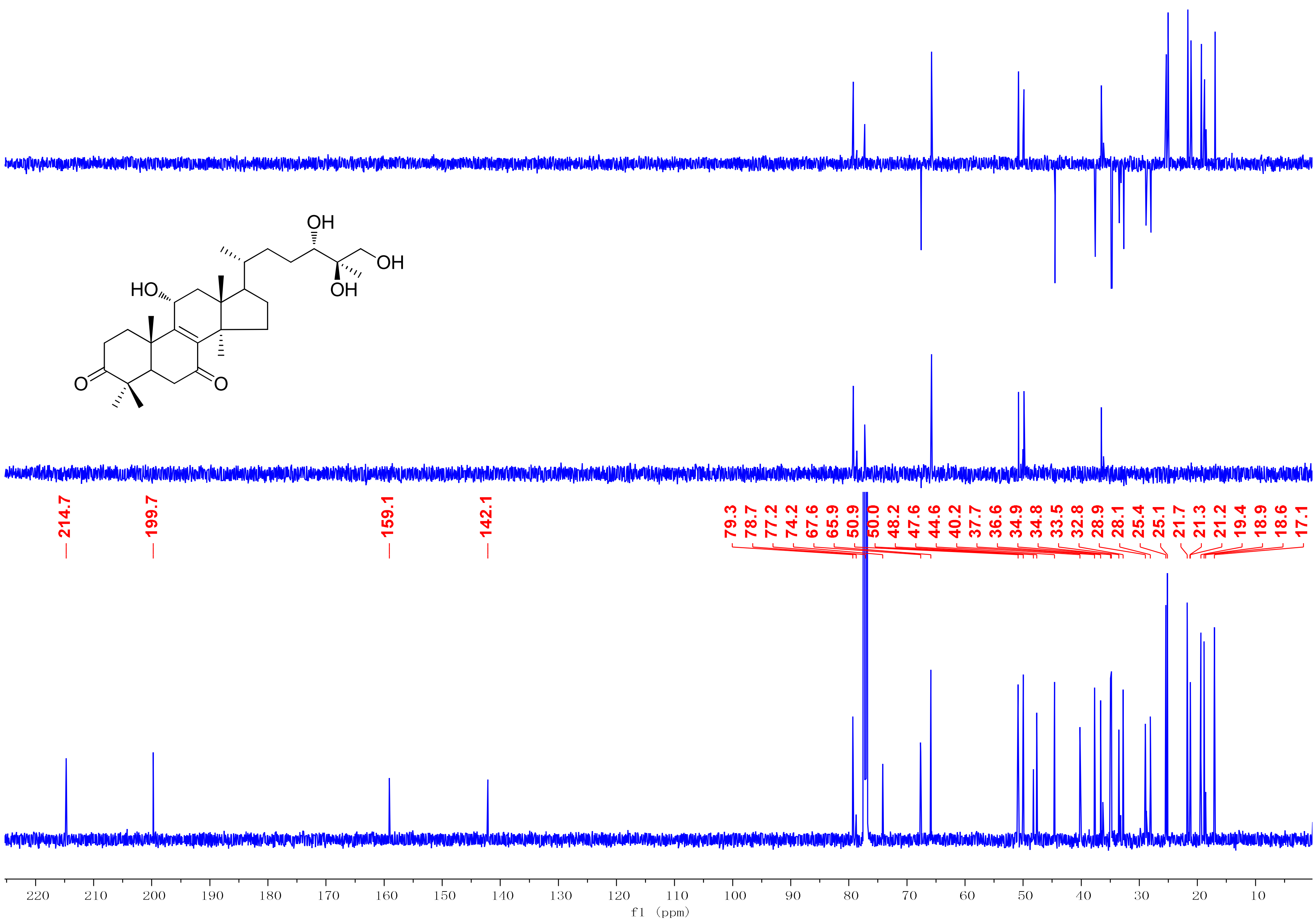

Figure S3. HSQC spectrum of compound **1** ($CDCl_3$).

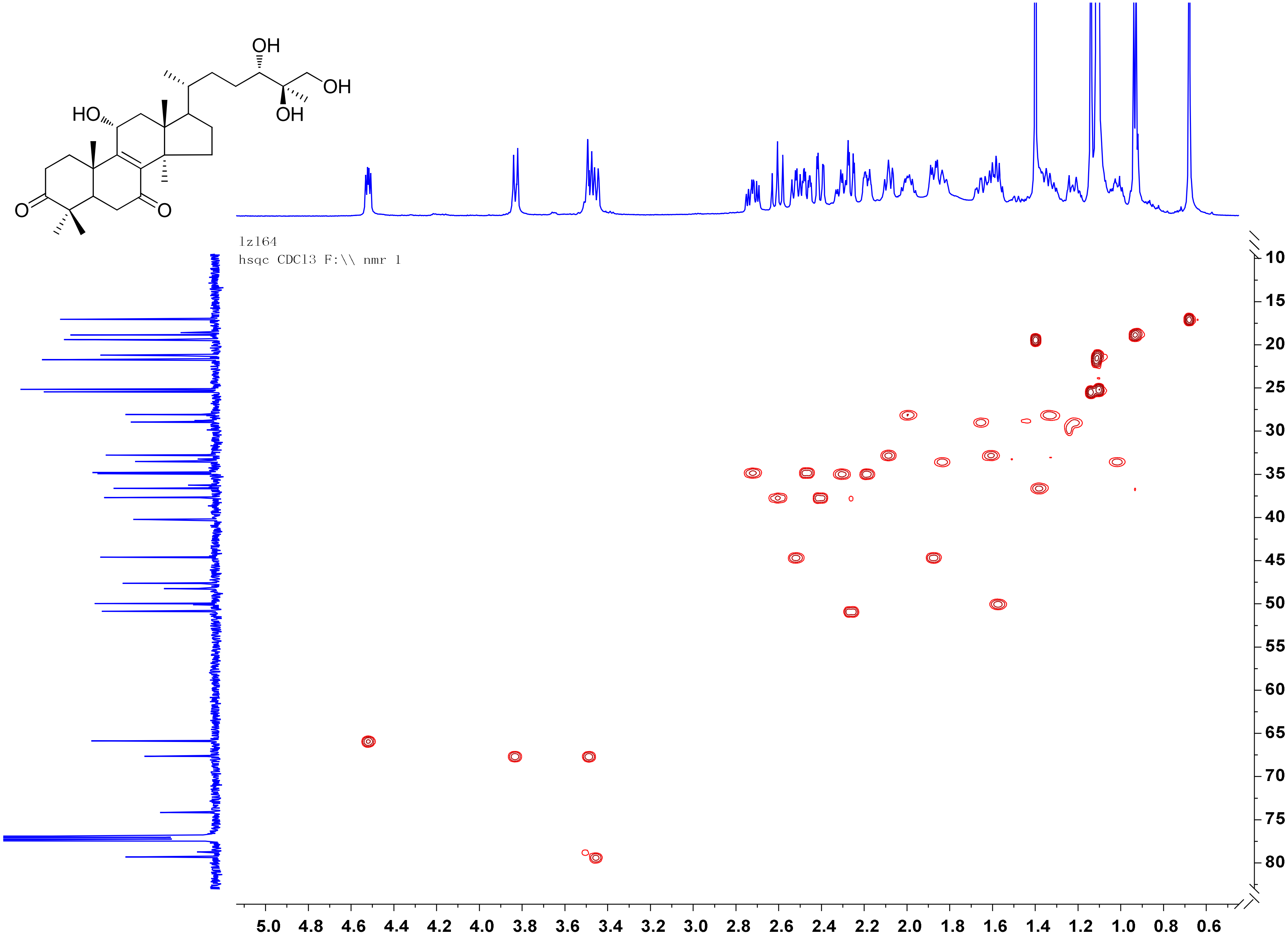

Figure S4. ^{1}H-^{1}H COSY spectrum of compound **1** ($CDCl_3$).

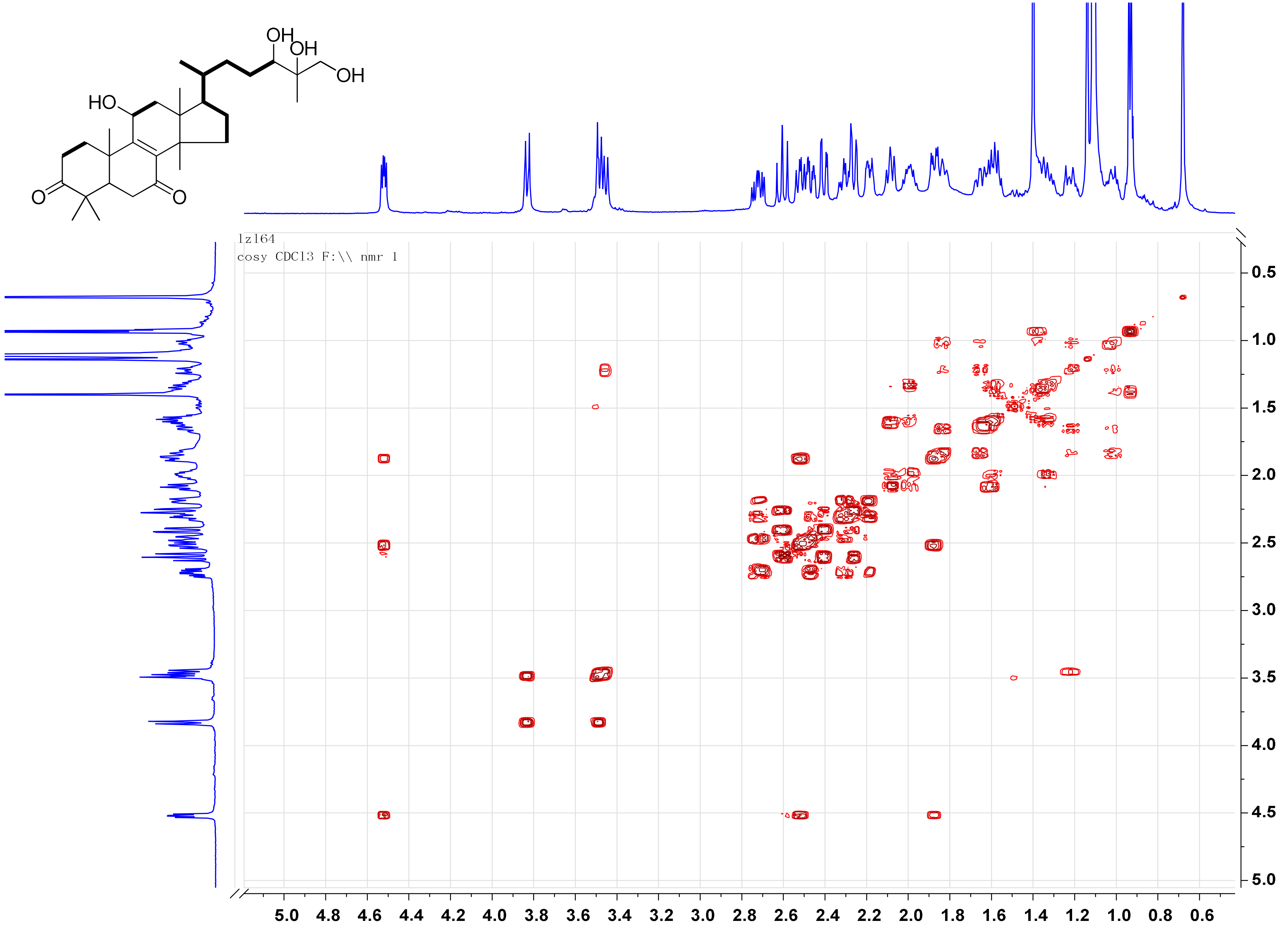

Figure S5. HMBC spectrum of compound **1** ($CDCl_3$).

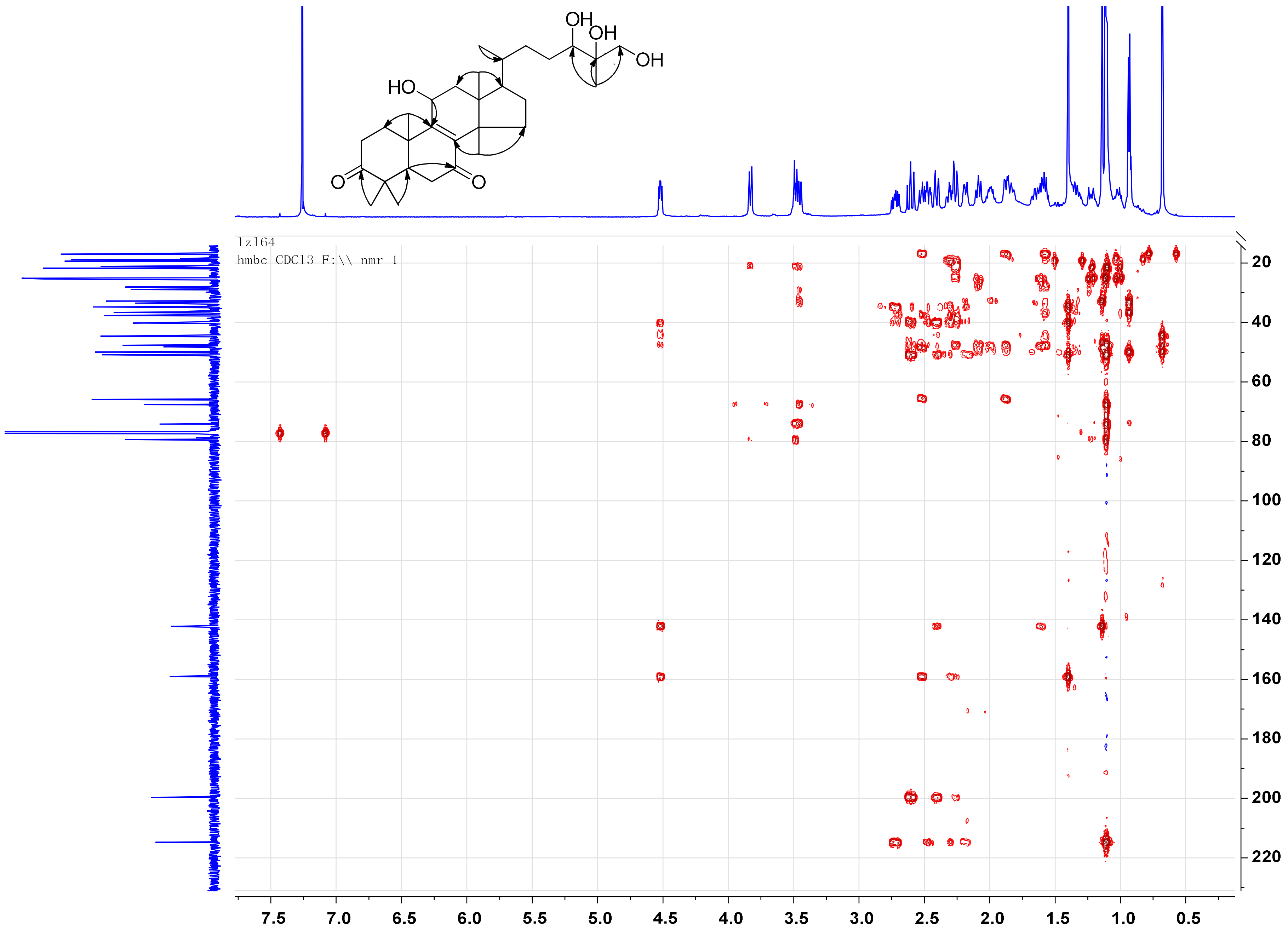

Figure S6. ROESY spectrum of compound **1** ($CDCl_3$).

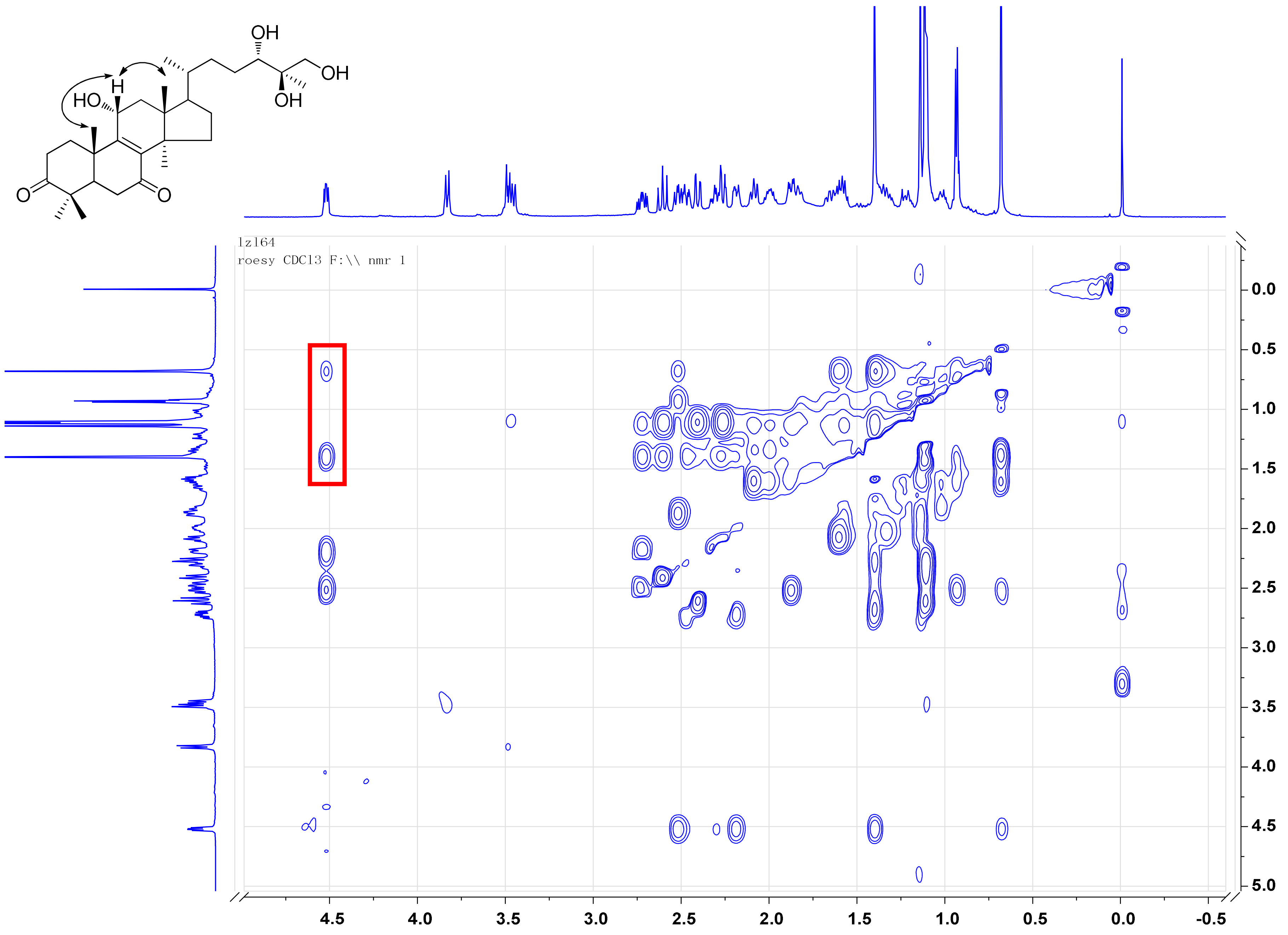

Figure S7. HREIMS spectrum of compound **1**.

Elemental Composition Report

Page 1

Single Mass Analysis

Tolerance = 10.0 PPM / DBE: min = -10.0, max = 120.0

Selected filters: None

Monoisotopic Mass, Odd and Even Electron Ions

21 formula(e) evaluated with 1 results within limits (up to 51 closest results for each mass)

Elements Used:

C: 0-200 H: 0-400 O: 4-6

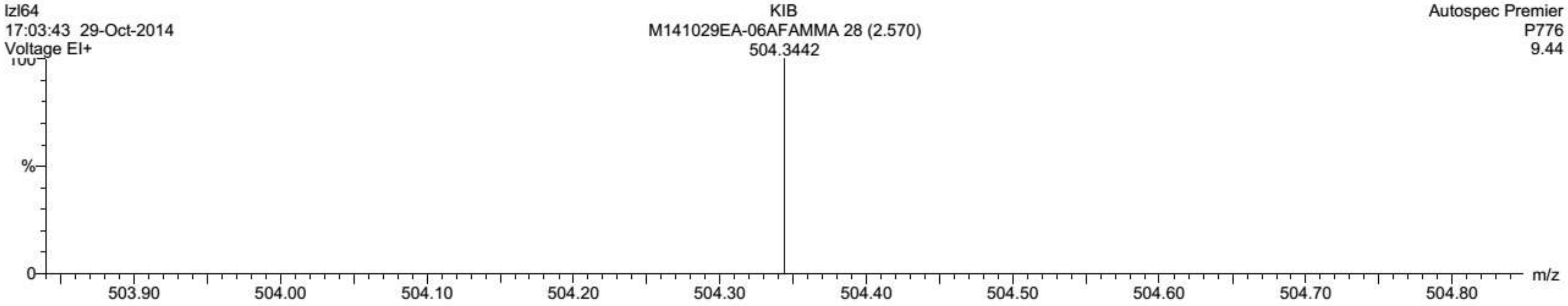

Minimum:				-10.0		
Maximum:		200.0	10.0	120.0		
Mass	Calc. Mass	mDa	PPM	DBE	i-FIT	Formula
504.3442	504.3451	-0.9	-1.8	7.0	5546026.5	C30 H48 O6

Figure S8. ^{1}H NMR spectrum of compound **2** ($CDCl_3$).

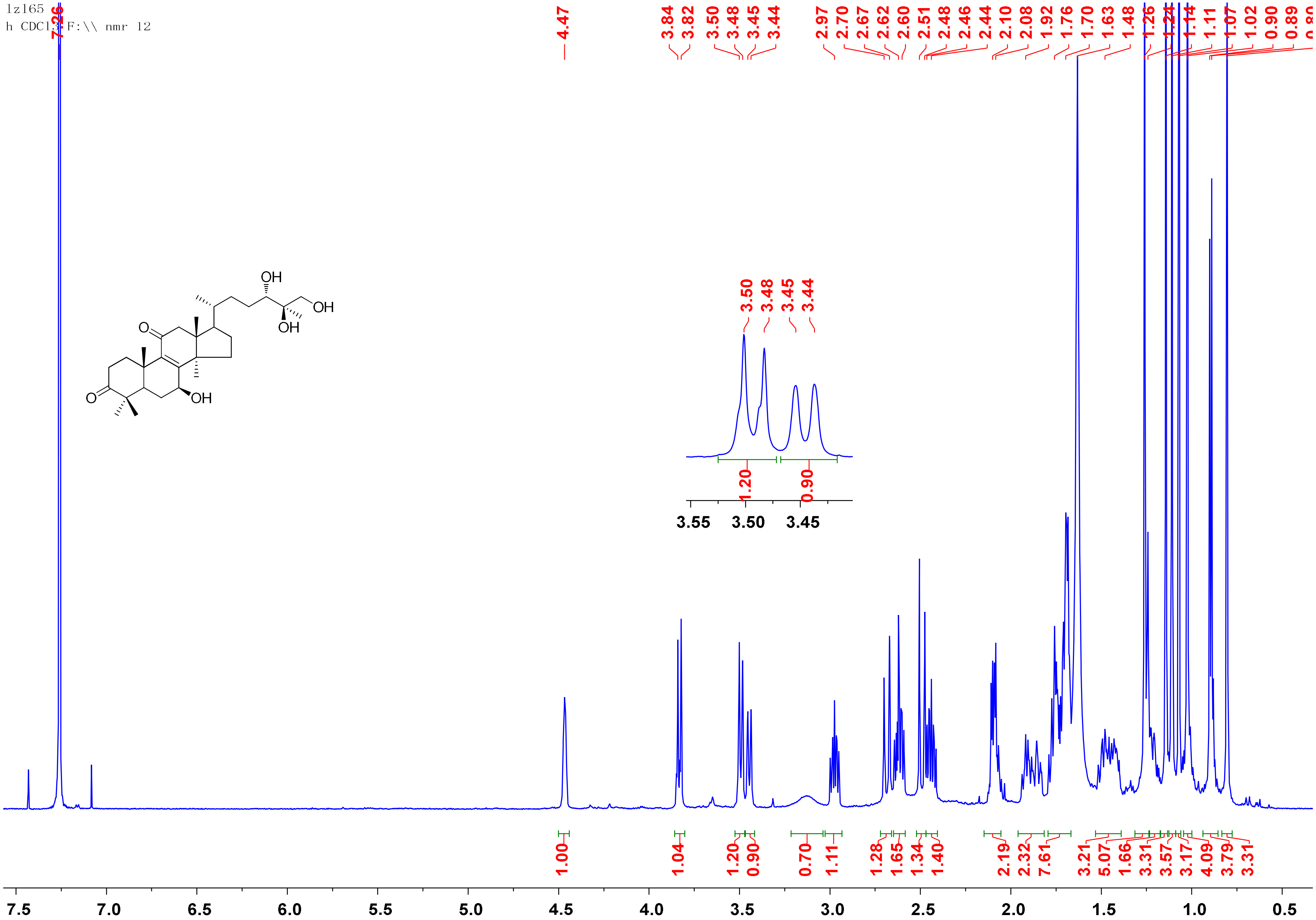

Figure S9. ^{13}C NMR and DEPT spectra of compound **2** ($CDCl_3$).

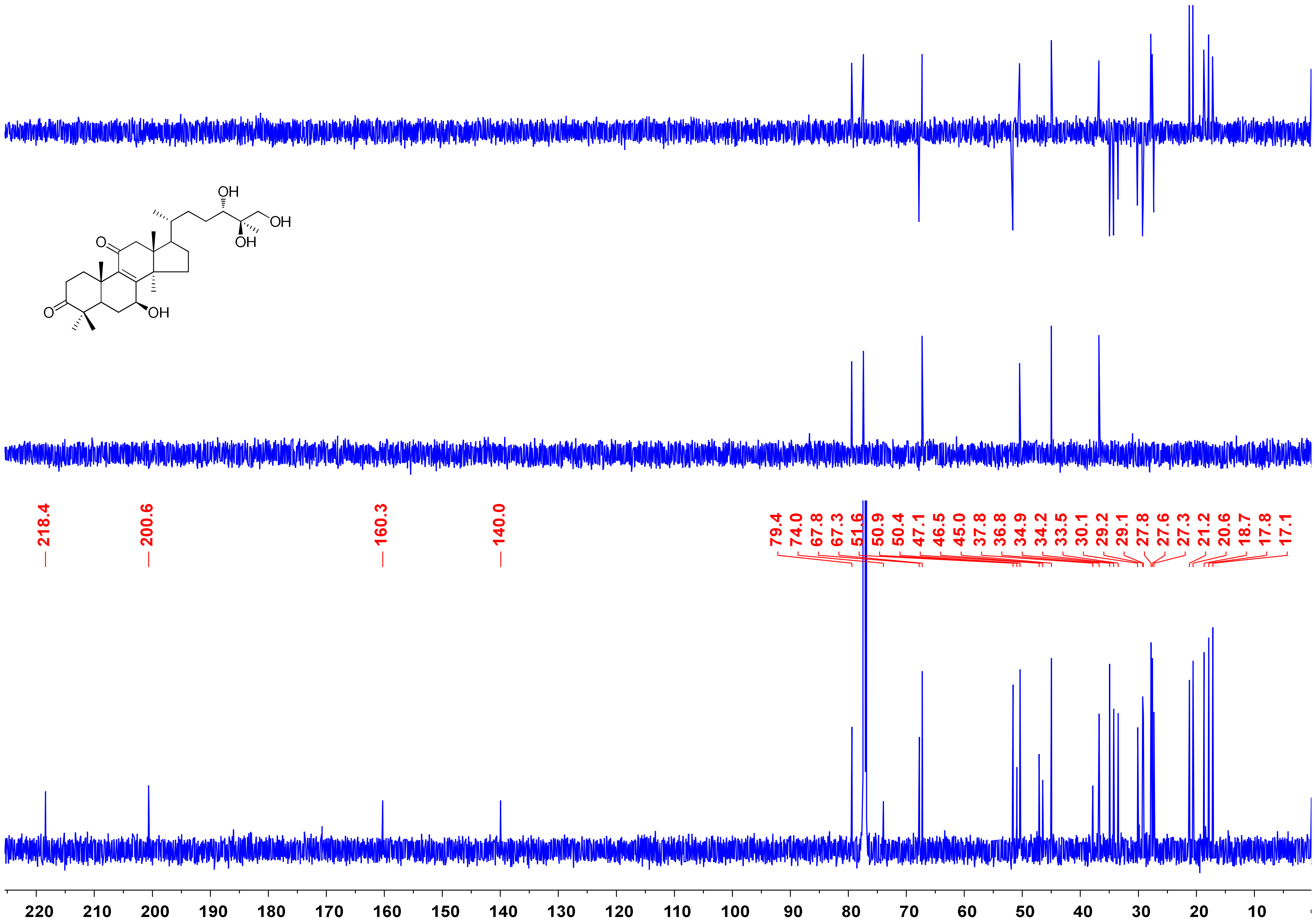

Figure S10. HSQC spectrum of compound **2** ($CDCl_3$).

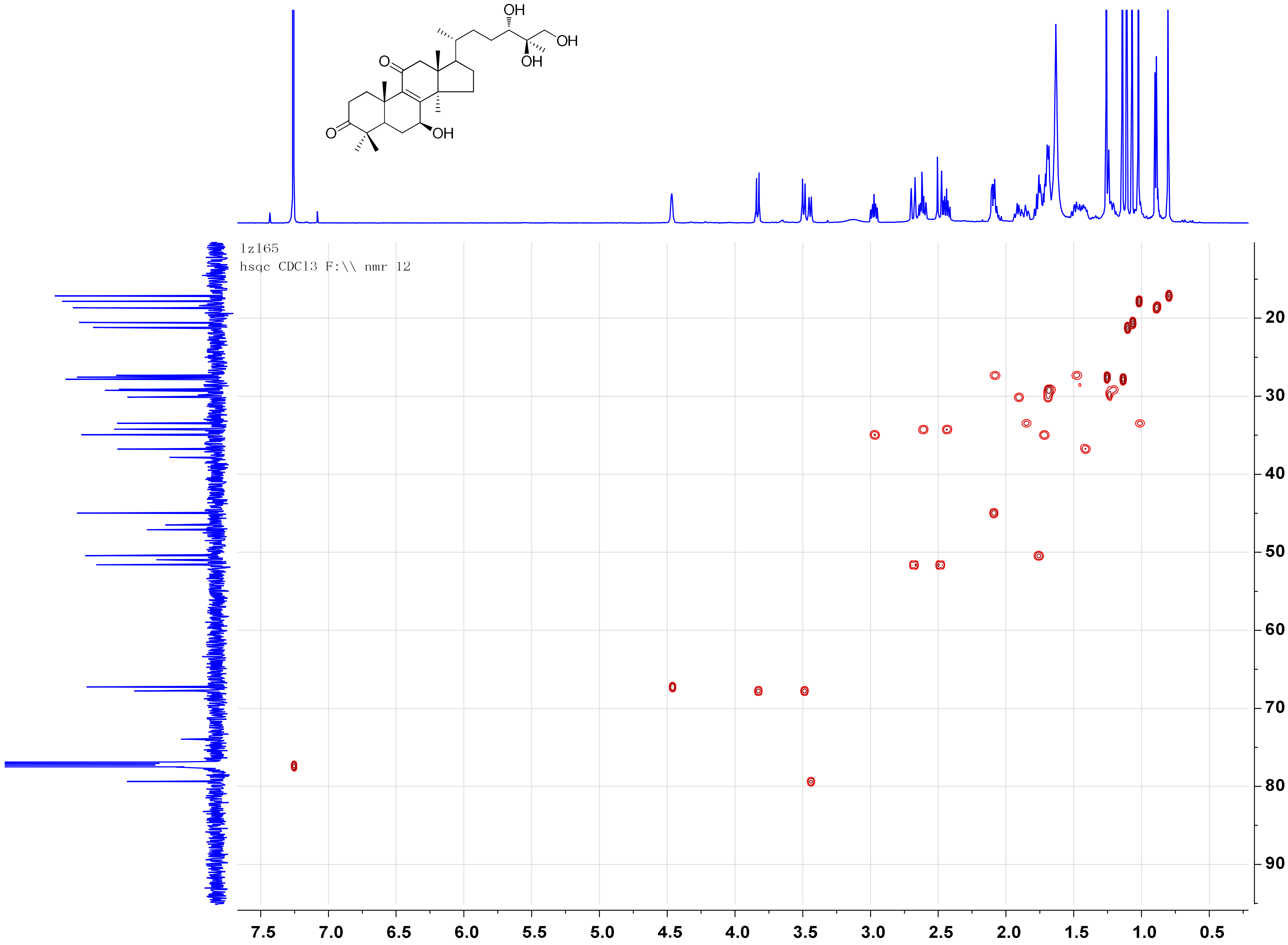

Figure S11. ^{1}H-^{1}H COSY spectrum of compound **2** ($CDCl_3$).

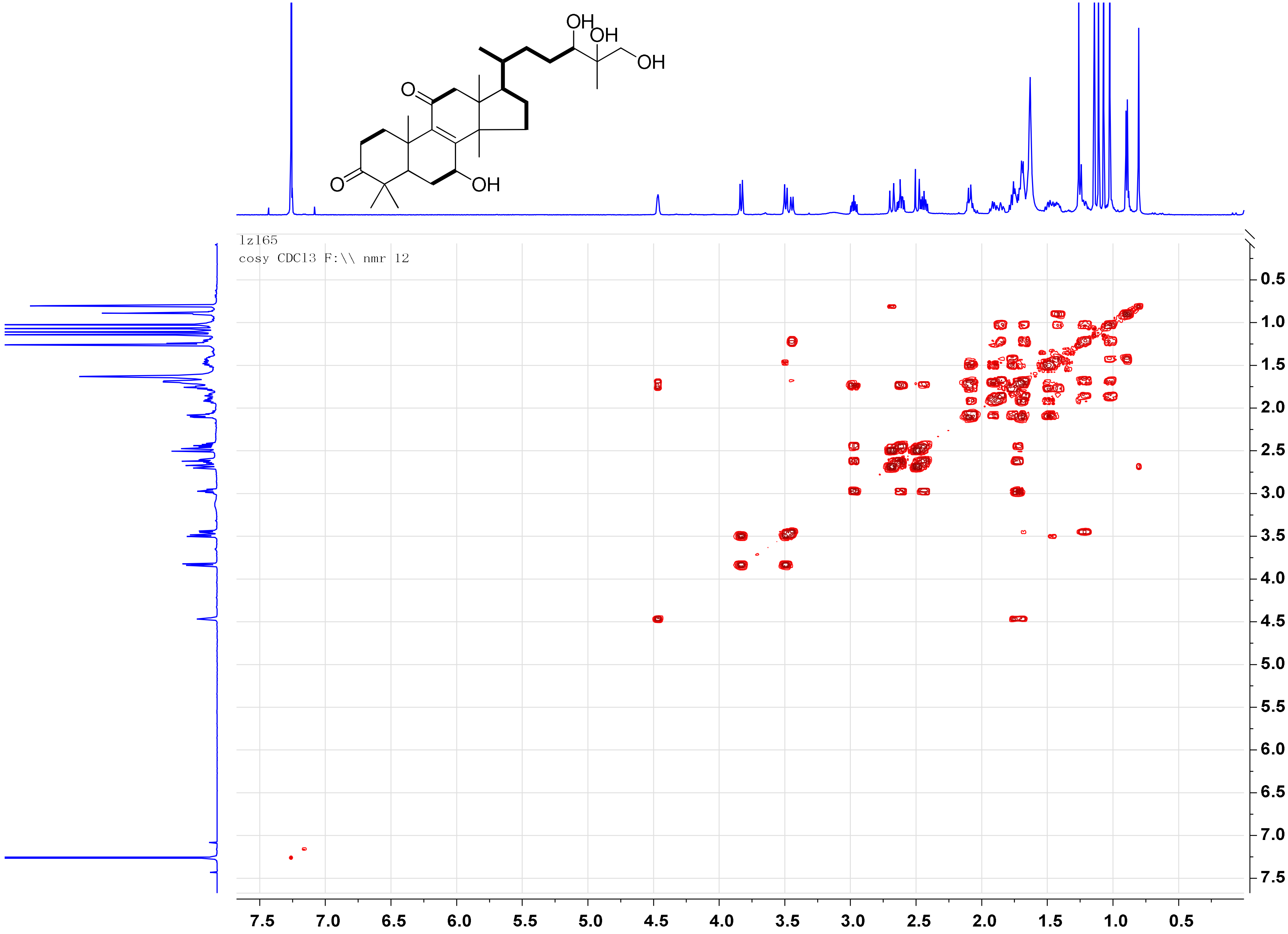

Figure S12. HMBC spectrum of compound **2** ($CDCl_3$).

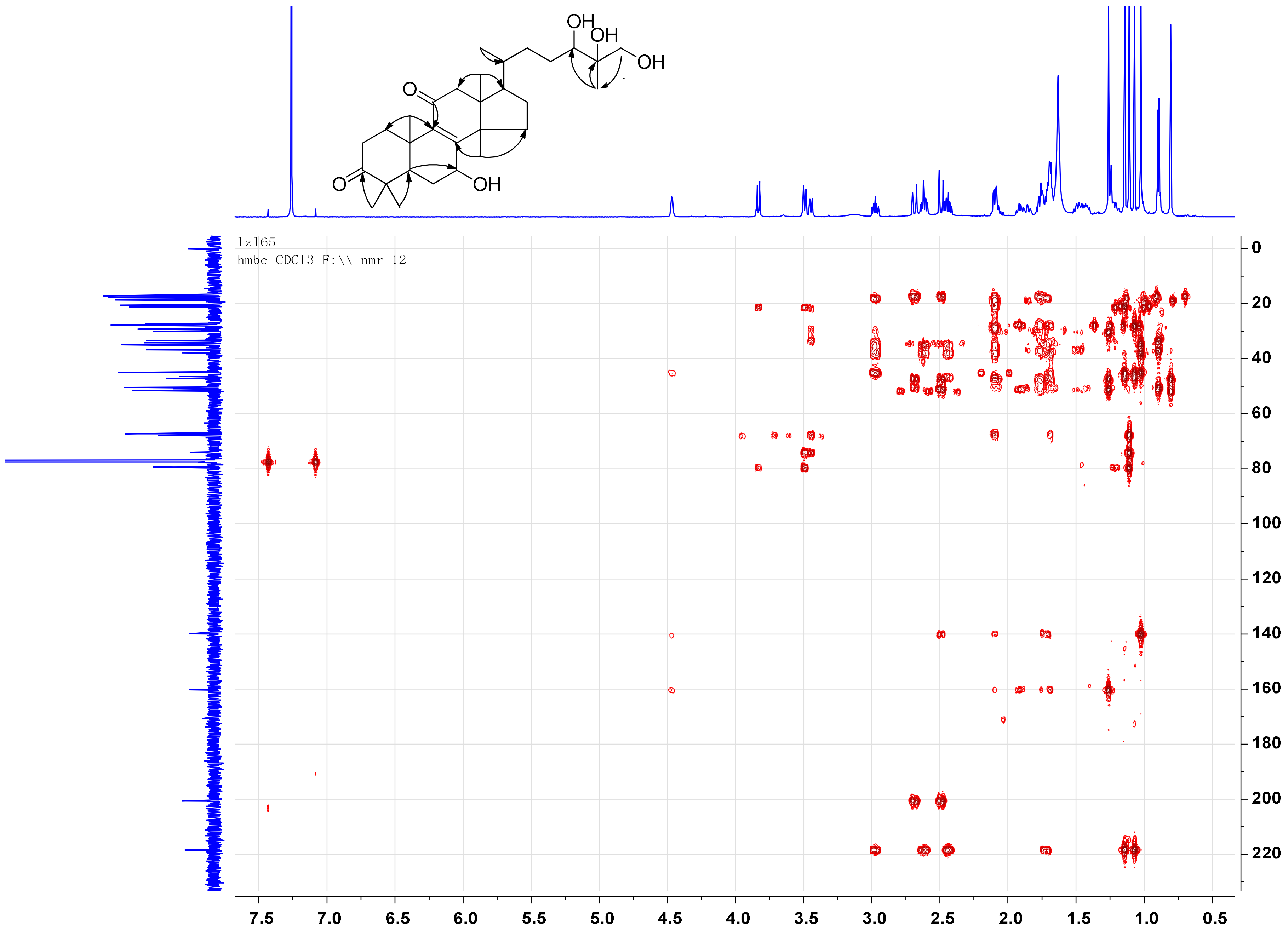

Figure S13. ROESY spectrum of compound **2** ($CDCl_3$).

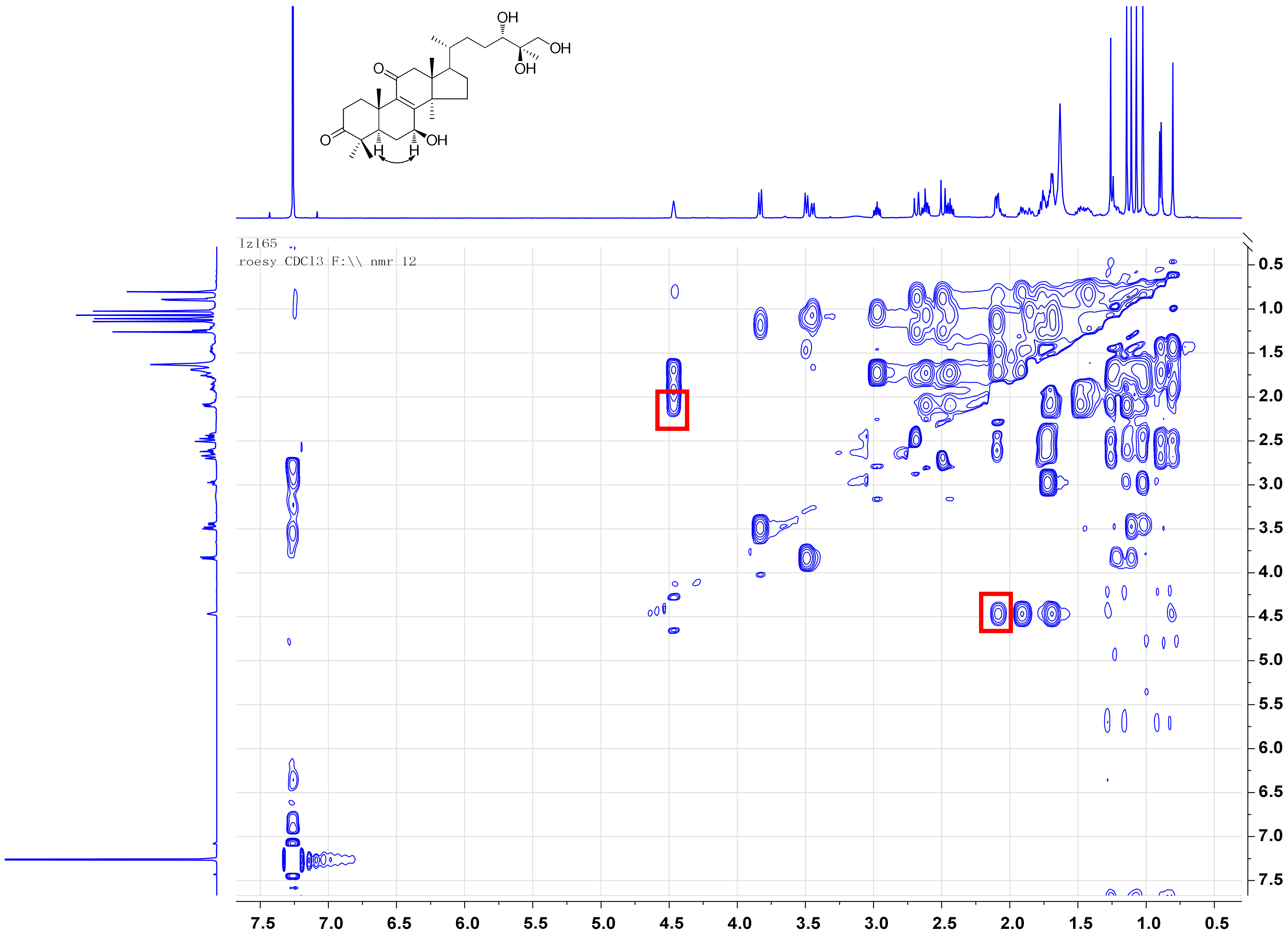

Figure S14. HREIMS spectrum of compound **2**.

Elemental Composition Report

Page 1

Single Mass Analysis

Tolerance = 10.0 PPM / DBE: min = -10.0, max = 120.0

Selected filters: None

Monoisotopic Mass, Odd and Even Electron Ions

21 formula(e) evaluated with 1 results within limits (up to 51 closest results for each mass)

Elements Used:

C: 0-200 H: 0-400 O: 4-6

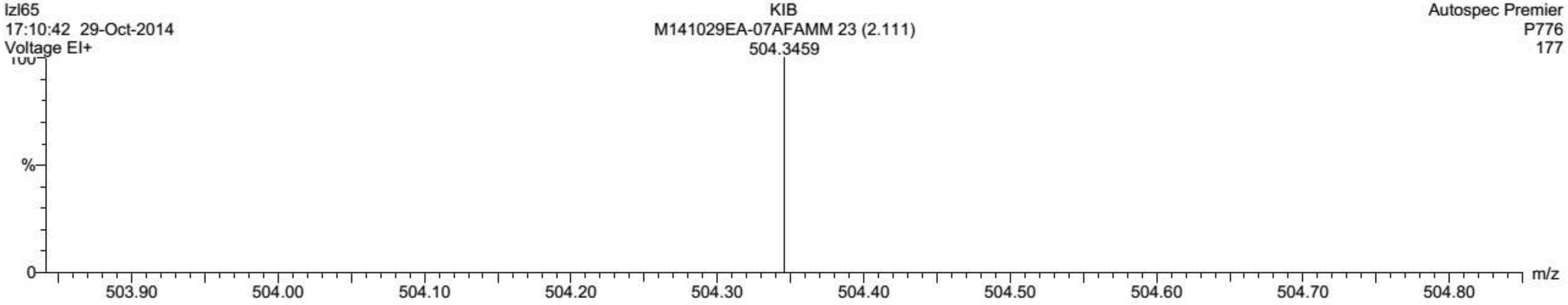

Mass	Calc. Mass	mDa	PPM	DBE	i-FIT	Formula
Minimum:				-10.0		
Maximum:		200.0	10.0	120.0		
504.3459	504.3451	0.8	1.6	7.0	5546106.5	C30 H48 O6

Figure S15. ^{1}H NMR spectrum of compound **3** (CD_3OD).

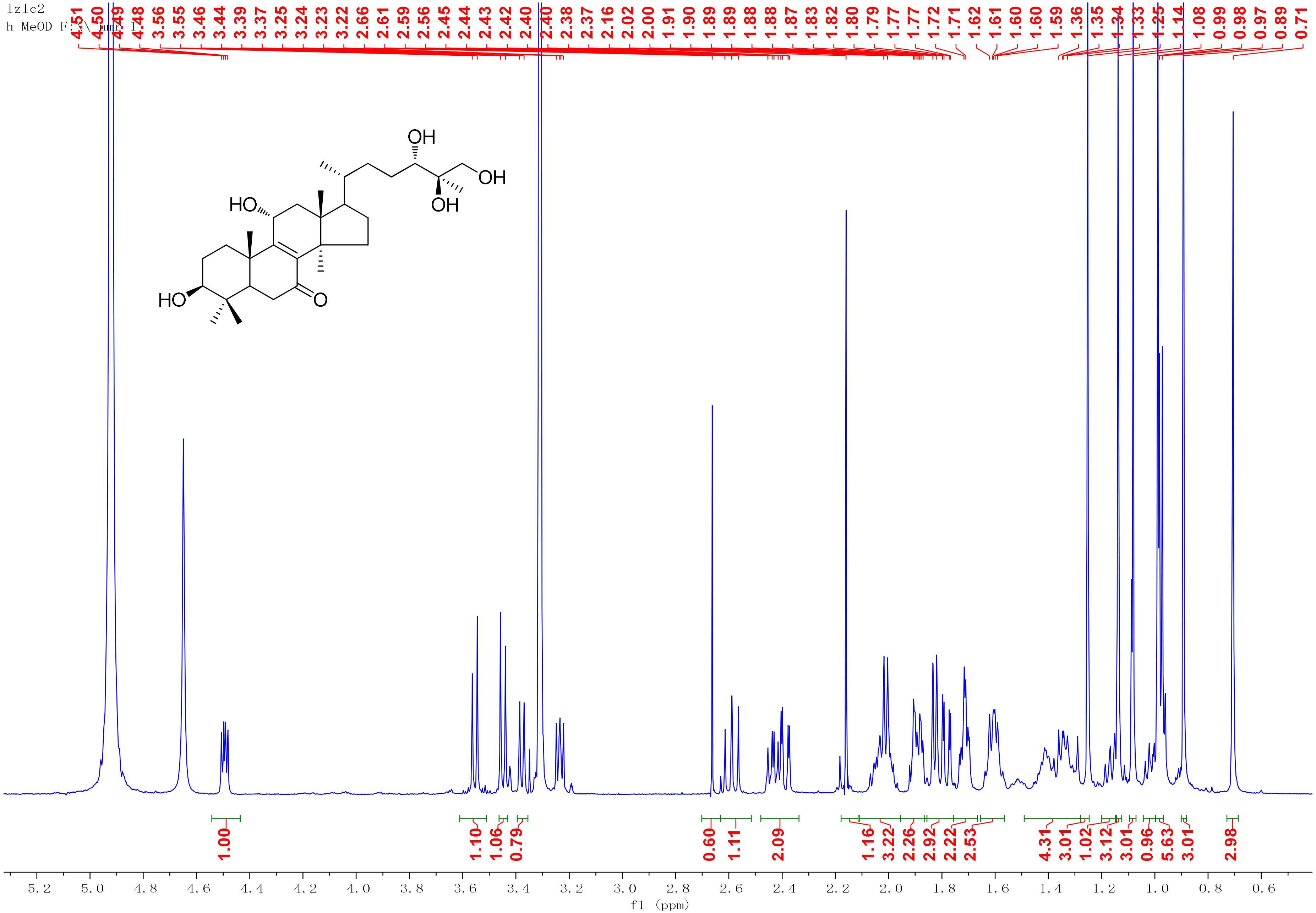

Figure S16. ^{13}C NMR and DEPT spectrum of compound **3** (CD_3OD)

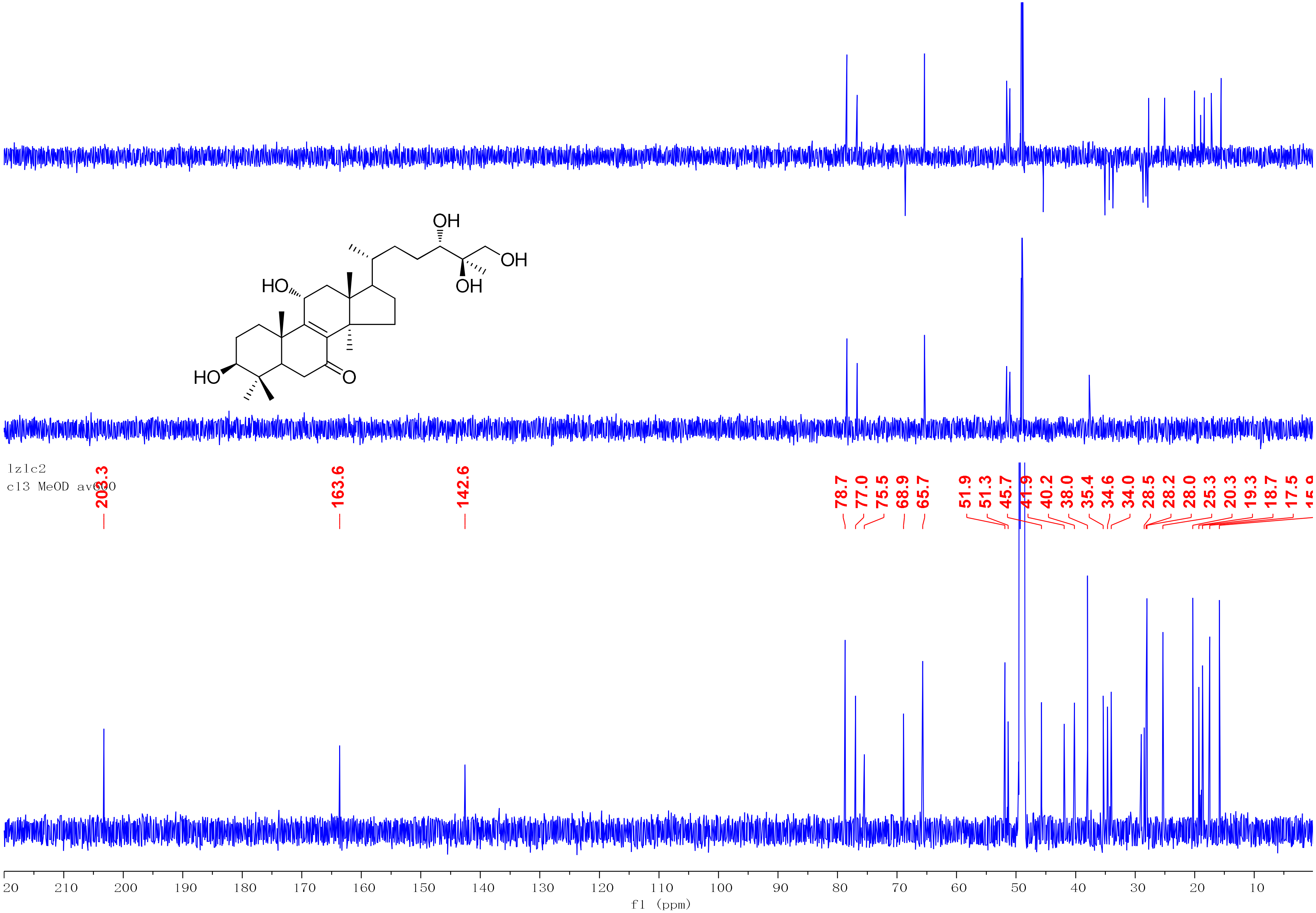

Figure S17. HSQC spectrum of compound **3** (CD_3OD).

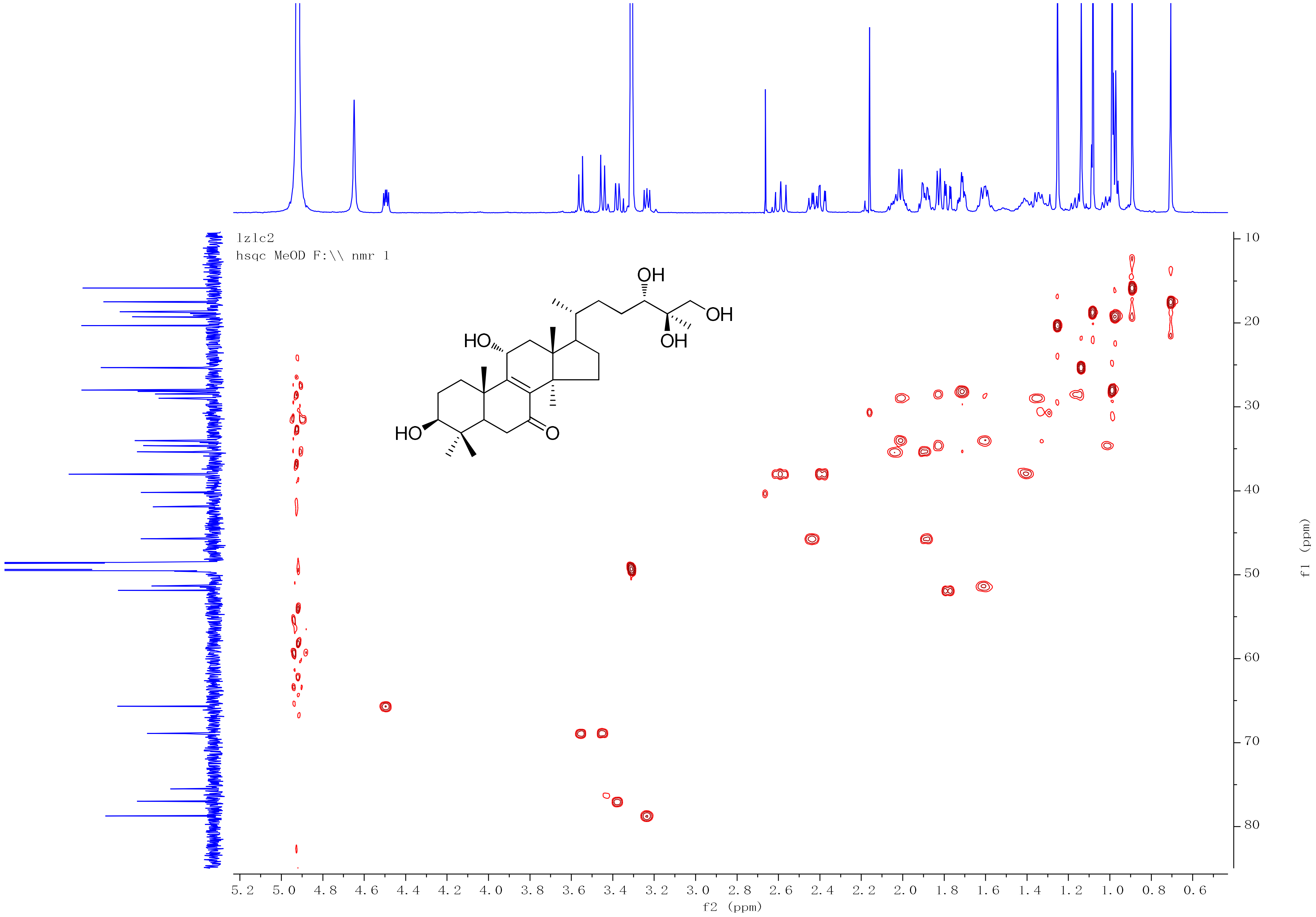

Figure S18. ^{1}H-^{1}H COSY spectrum of compound **3** (CD_3OD).

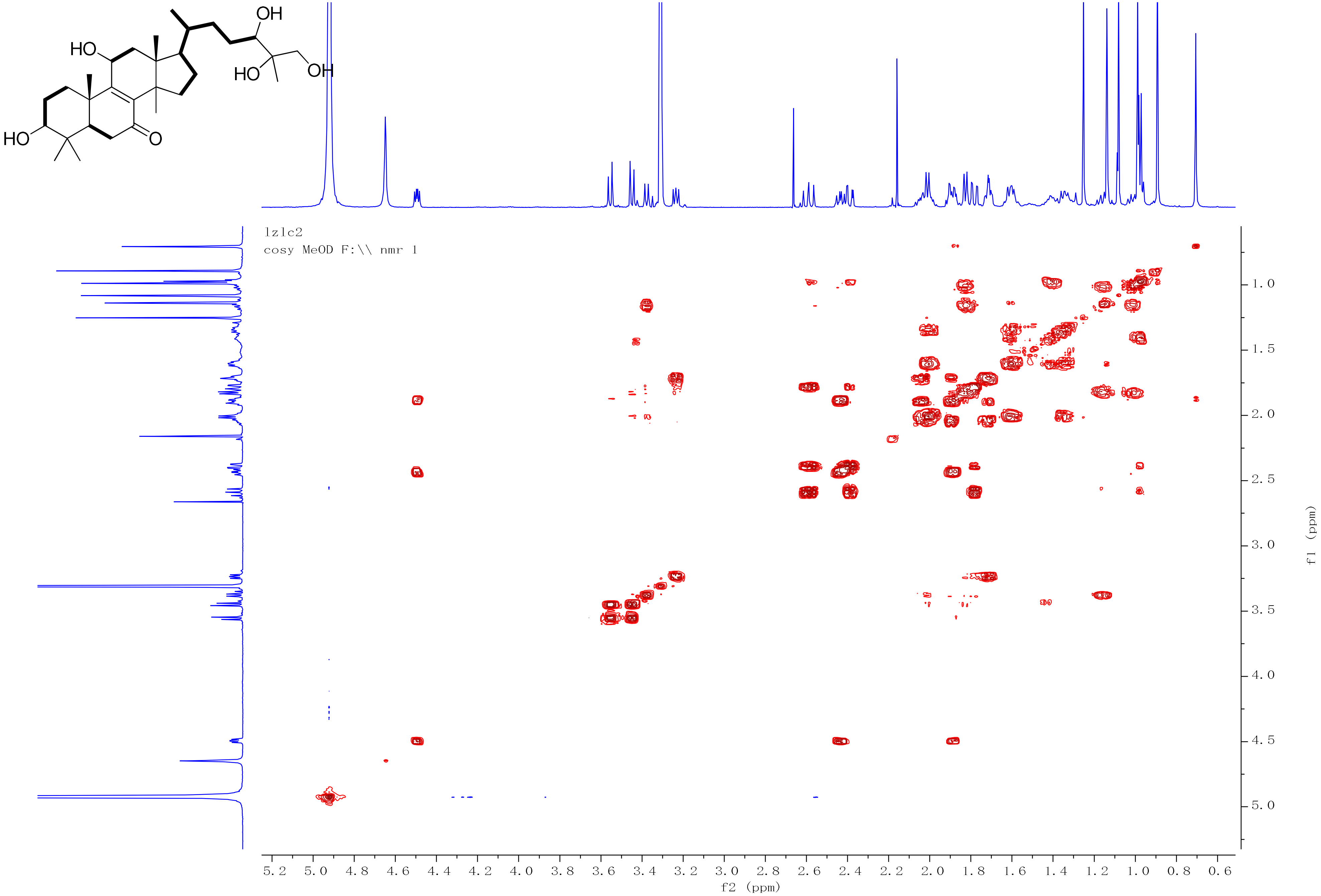

Figure S19. HMBC spectrum of compound **3** (CD_3OD).

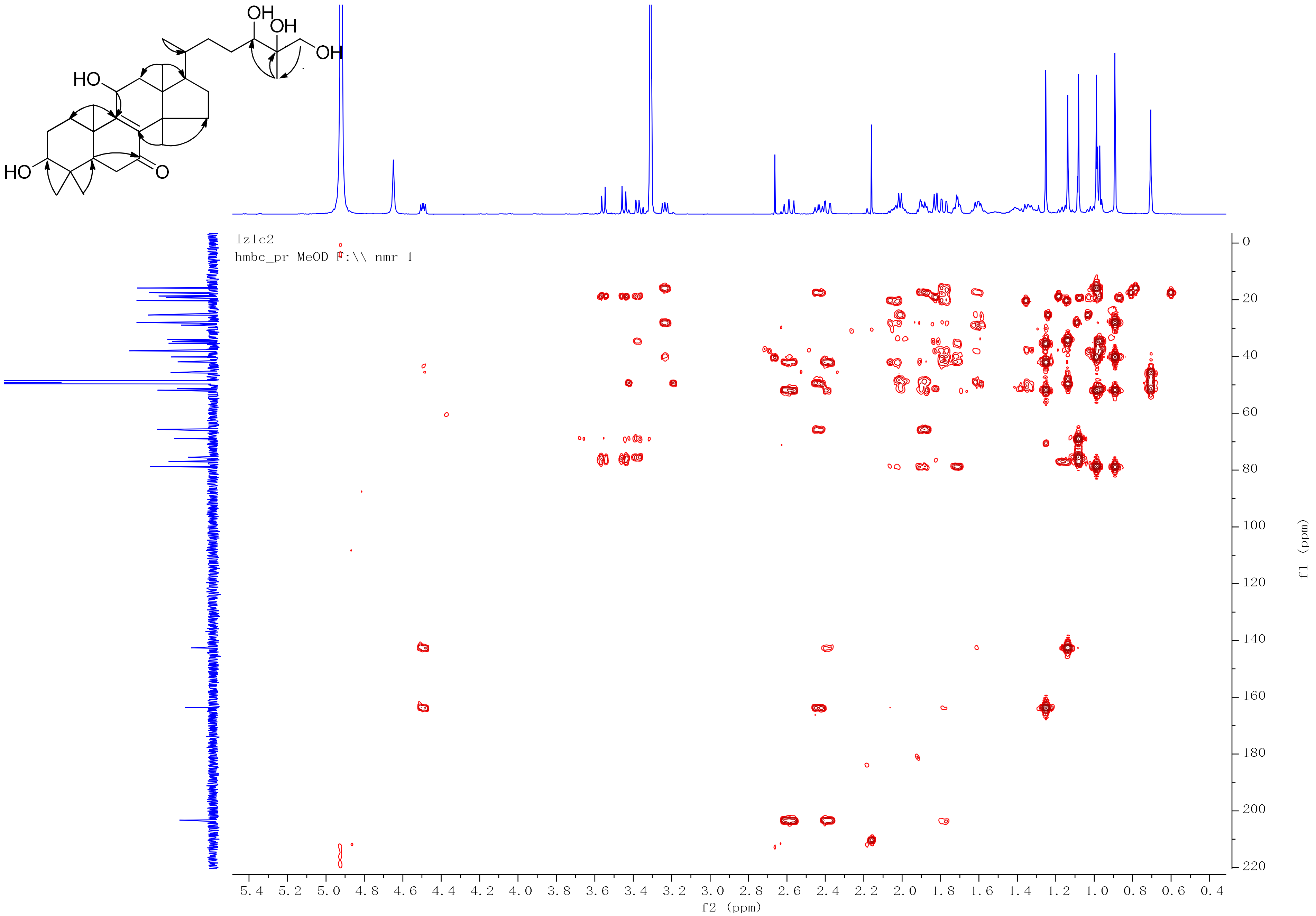

Figure S20. ROESY spectrum of compound **3** (CD_3OD).

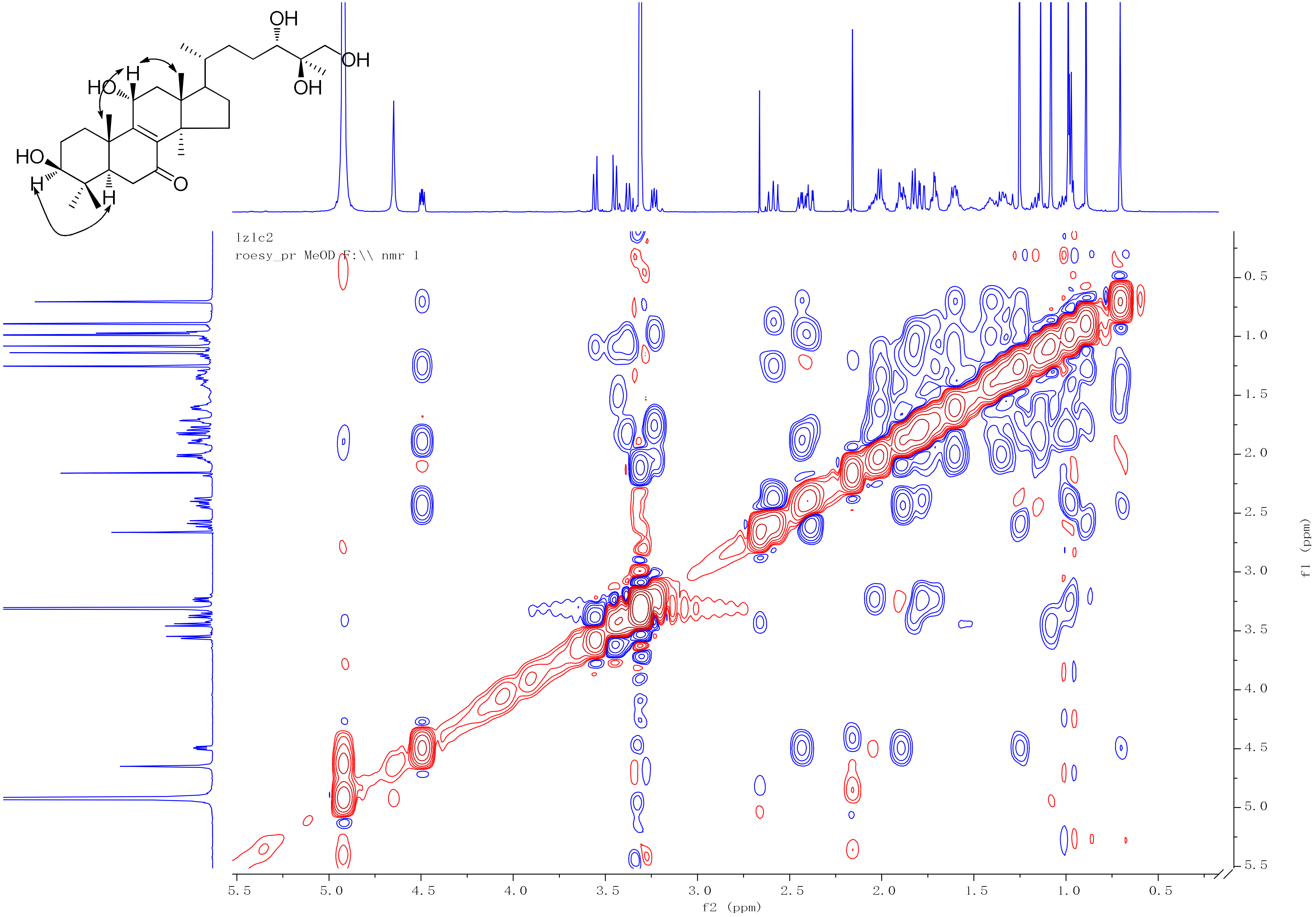

Figure S21. HRESIMS spectrum of compound **3**.

Qualitative Analysis Report

**Data Filename**	LZL-C2.d	**Sample Name**	LZL-C2
**Sample Type**	Sample	**Position**	P1-B1
**Instrument Name**	Instrument 1	**User Name**	
**Acq Method**	SIBU.m	**Acquired Time**	3/30/2015 2:07:01 PM
**IRM Calibration Status**	Success	**DA Method**	Default.m
**Comment**			

Sample Group **Info.**

Acquisition SW 6200 series TOF/6500 series

Version Q-TOF B.05.01 (B5125.2)

User Spectra

Fragmentor Voltage	Collision Energy	Ionization Mode
135	0	ESI

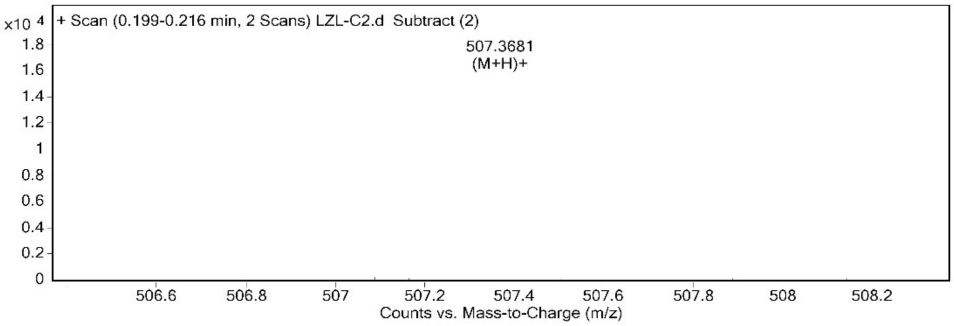

Peak List

m/z	z	Abund	Formula	Ion
274.2741	1	15577.36		
318.3	1	20043.65		
507.3681	1	15957.93	C30 H50 O6	(M+H)+
576.4101	1	13824.36		
620.4367	1	16326		
664.4628	1	13485.24		

Formula Calculator Element Limits

Element	Min	Max
C	3	60
H	0	120
O	0	30

Formula Calculator Results

Formula	CalculatedMass	CalculatedMz	Mz	Diff. (mDa)	Diff. (ppm)	DBE
C30 H50 O6	506.3607	507.3680	507.3681	-0.3	-0.6	6.0000

--- End Of Report ---

Figure S22. ^{1}H NMR spectrum of compound **4** ($CDCl_3$).

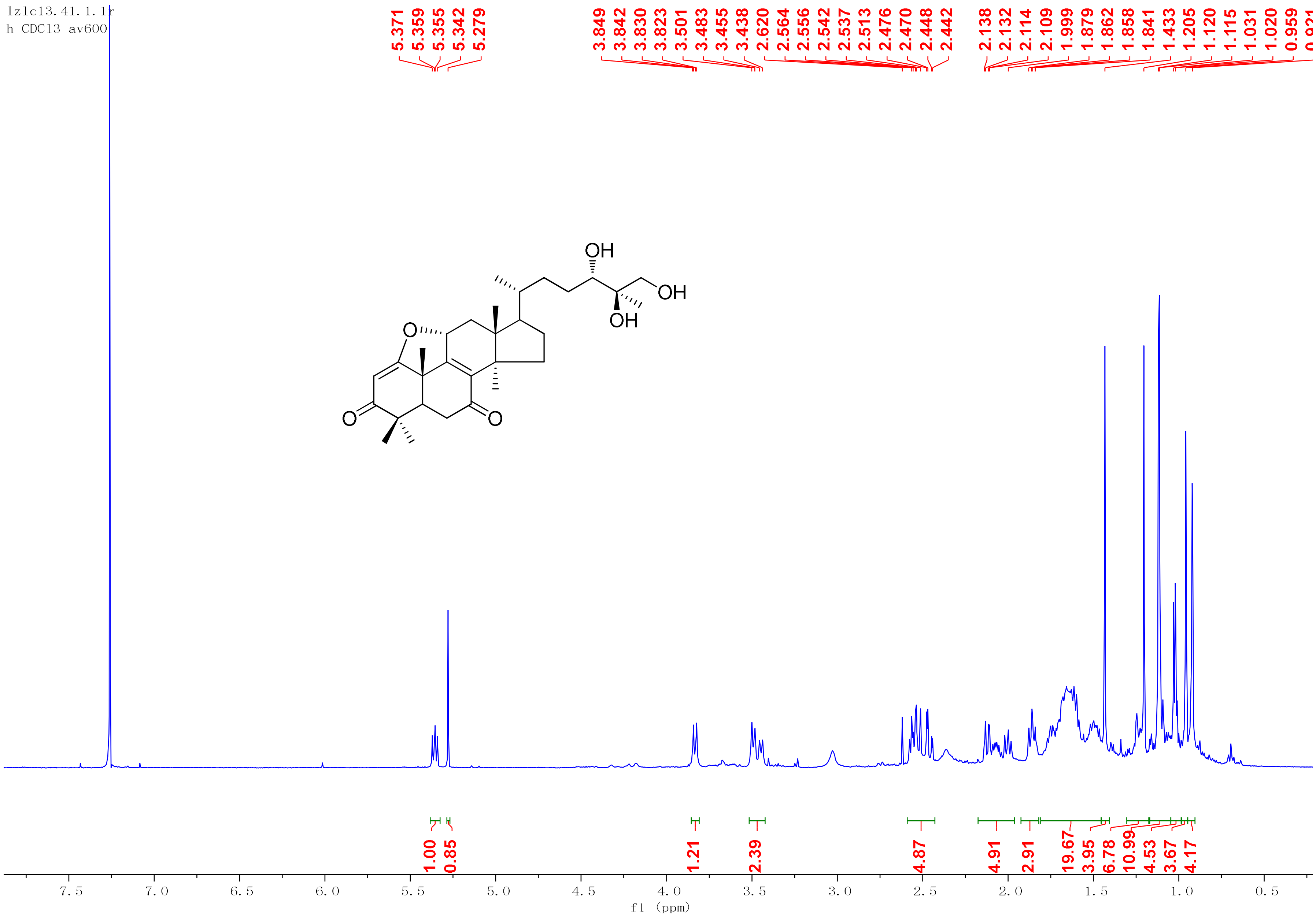

Figure S23. ^{13}C NMR and DEPT spectra of compound **4** ($CDCl_3$).

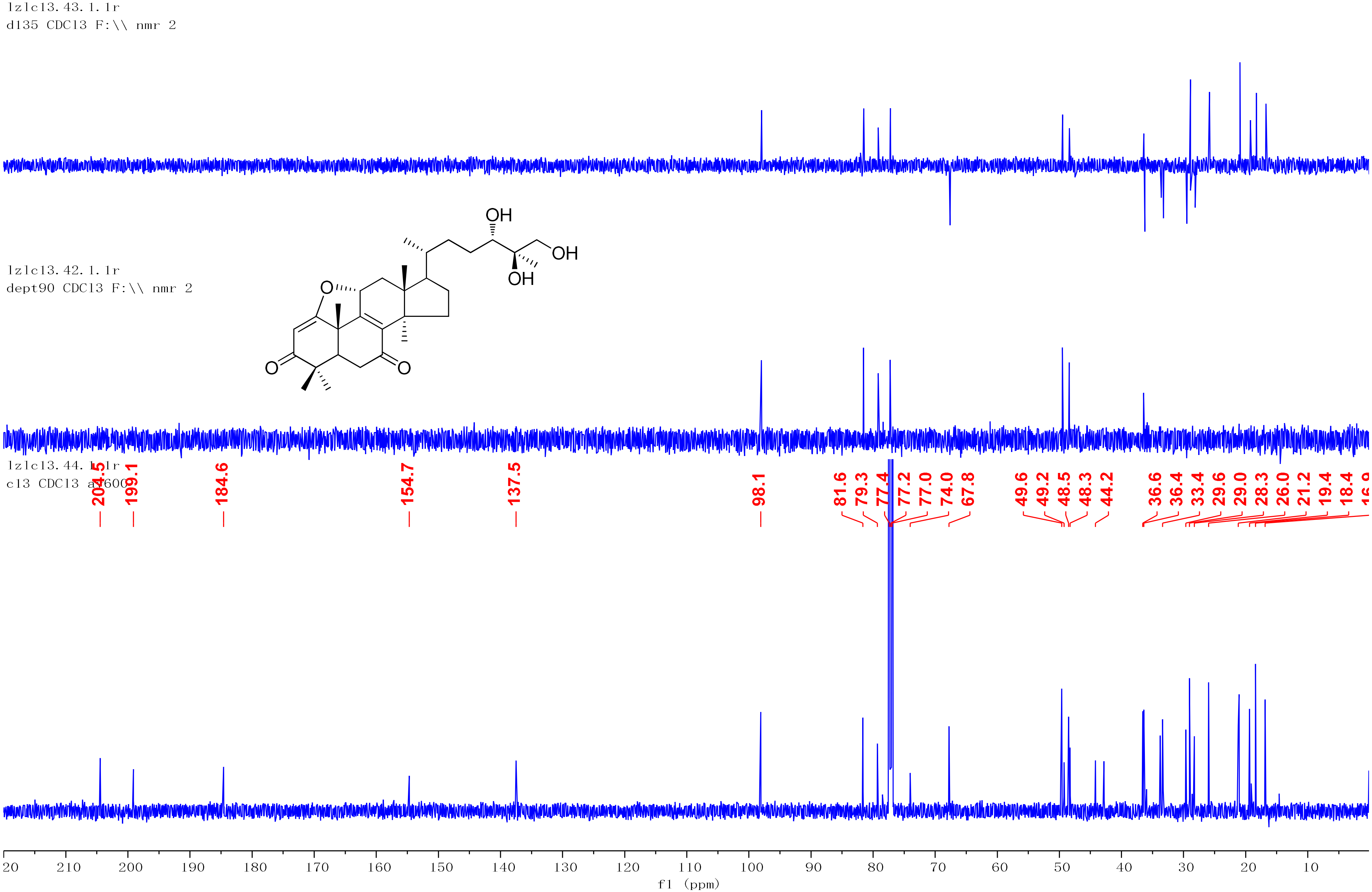

Figure S24. HSQC spectrum of compound **4** ($CDCl_3$).

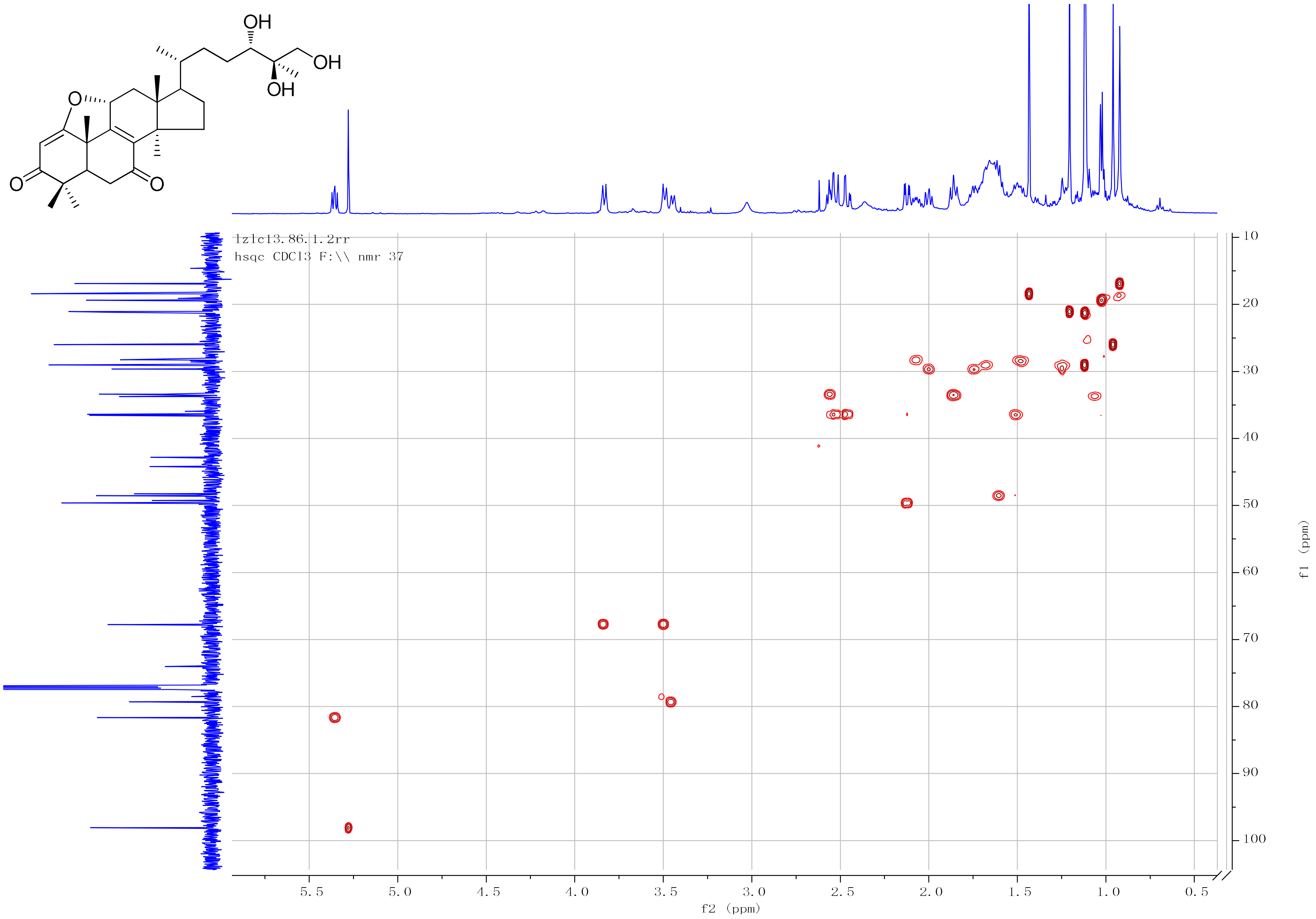

Figure S25. ^{1}H-^{1}H COSY spectrum of compound **4** ($CDCl_3$).

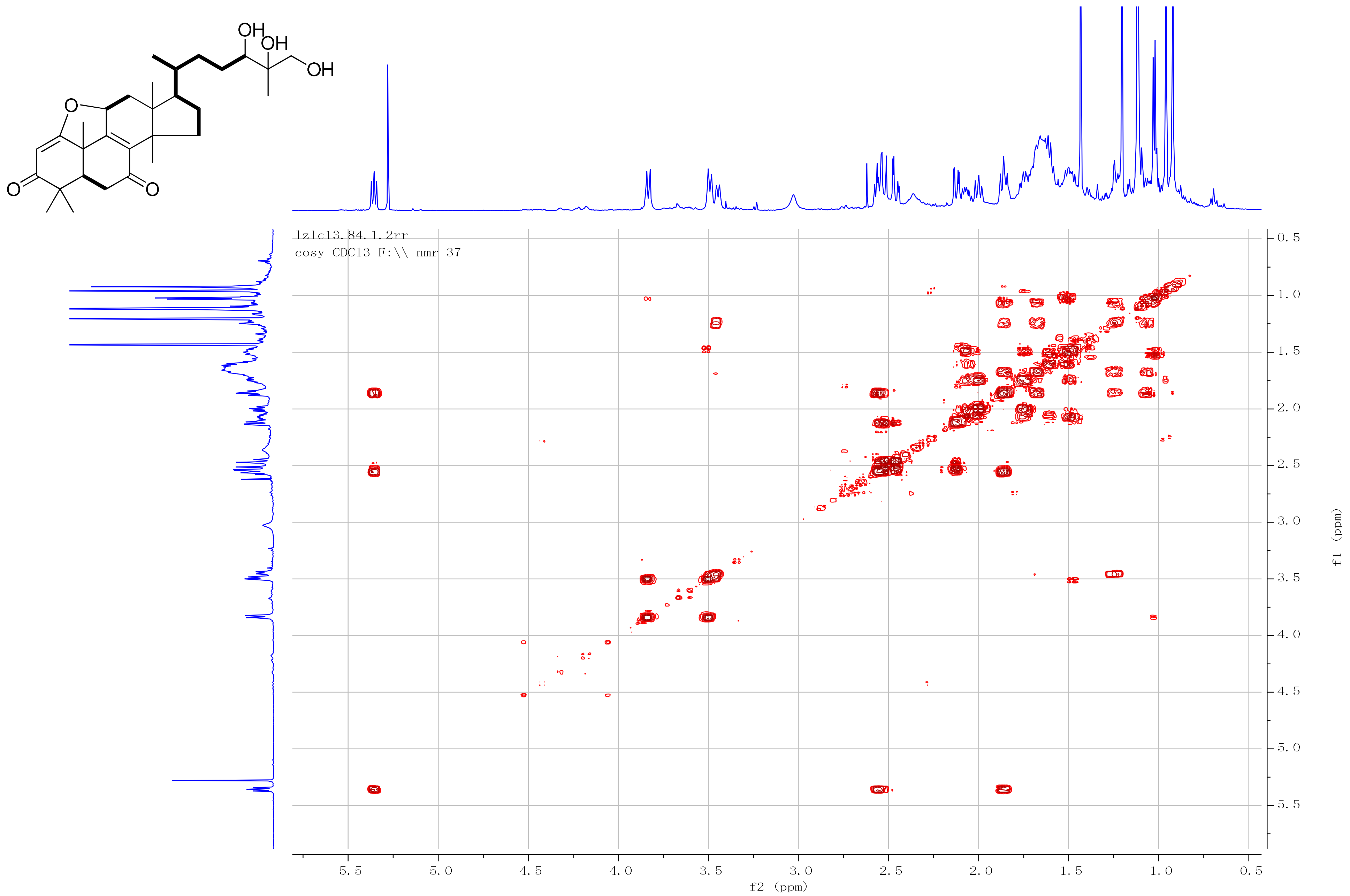

Figure S26. HMBC spectrum of compound **4** ($CDCl_3$).

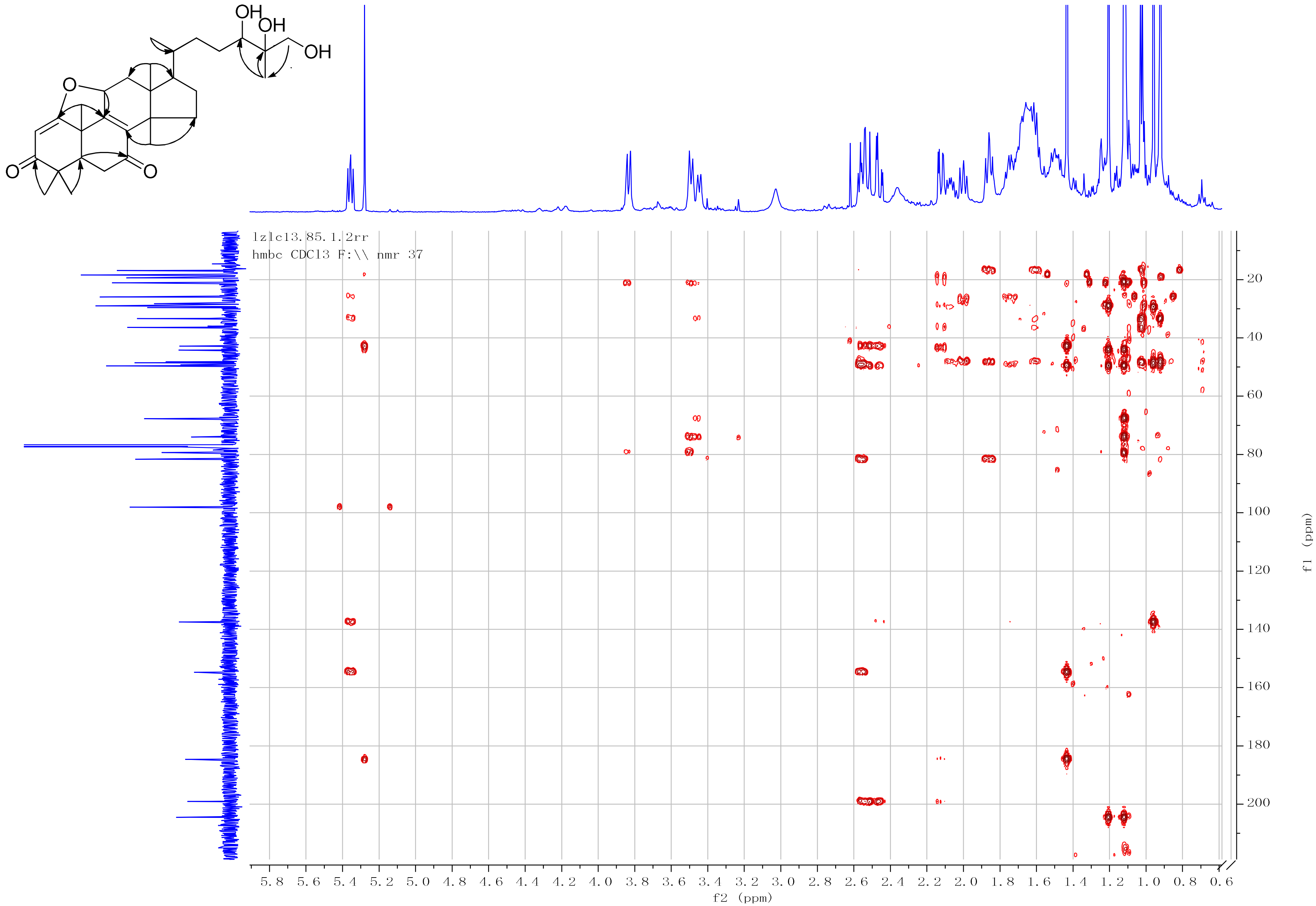

Figure S27. ROESY spectrum of compound **4** ($CDCl_3$).

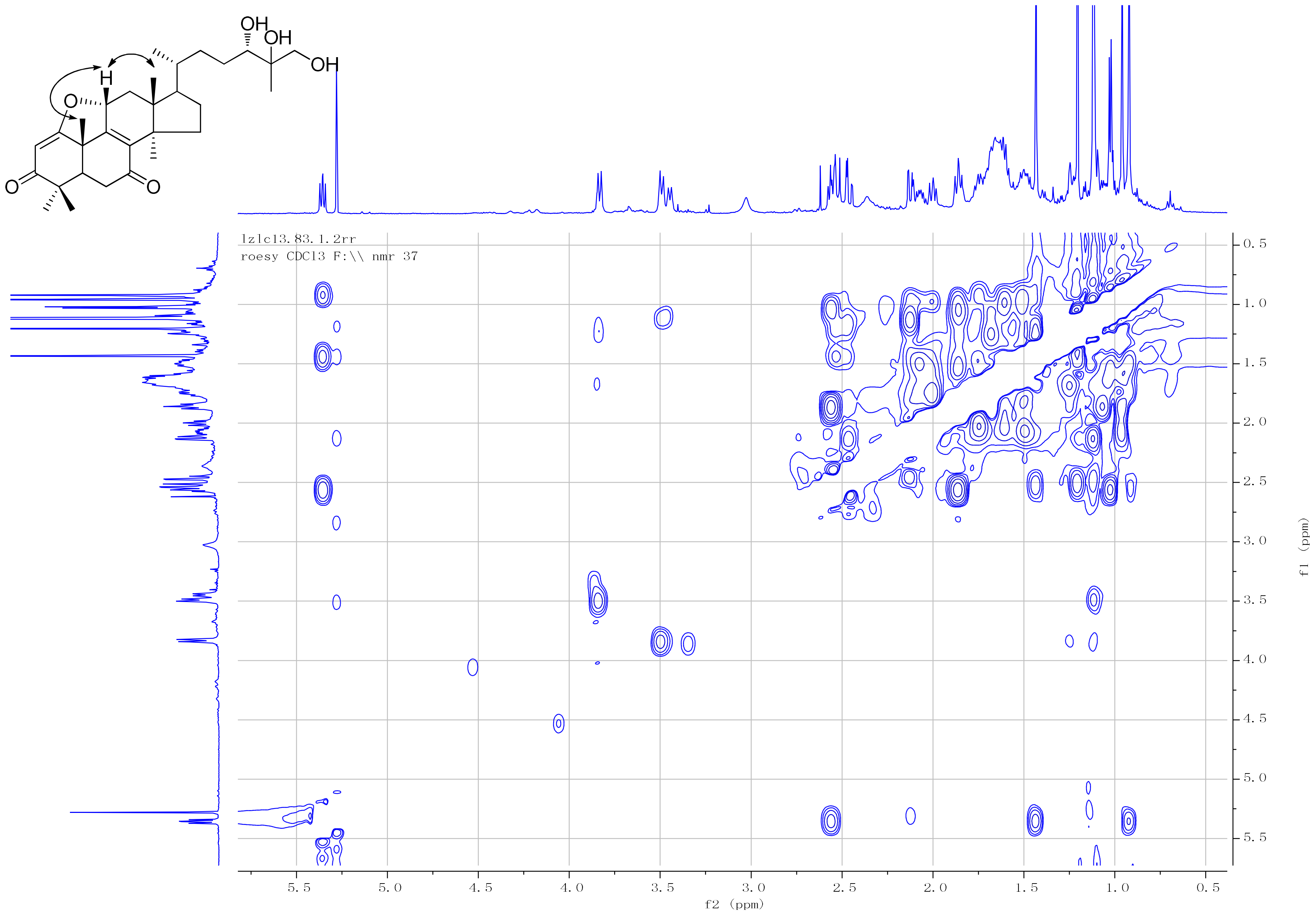

Figure S28. HREIMS spectrum of compound **4**.

Qualitative Analysis Report

**Data Filename**	LZL-C13.d	**Sample Name**	LZL-C13
**Sample Type**	Sample	**Position**	P1-A7
**Instrument Name**	Instrument 1	**User Name**	
**Acq Method**	SIBU.m	**Acquired Time**	3/30/2015 2:01:23 PM
**IRM Calibration Status**	Success	**DA Method**	Default.m
**Comment**			

Sample Group **Info.**
Acquisition SW Version 6200 series TOF/6500 series Q-TOF B.05.01 (B5125.2)

User Spectra

Fragmentor Voltage	Collision Energy	Ionization Mode
135	0	ESI

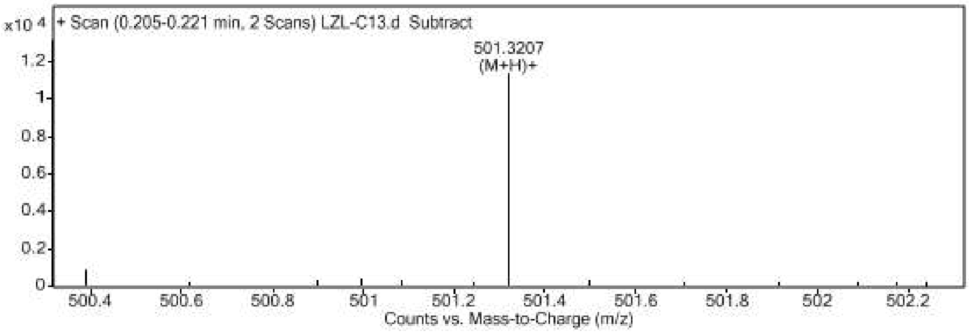

Peak List

m/z	z	Abund
98.9753		50134.13
488.3577	1	39634.05
532.3842	1	72225.2
553.3136	1	31097.37
576.4103	1	86678.87
577.4137	1	27360.77
597.3395	1	35322.77
620.4366	1	68038.23
641.3657	1	27755.39
664.4626	1	45232.21

Formula Calculator Element Limits

Element	Min	Max
C	3	60
H	0	120
O	0	30

Formula Calculator Results

Formula	CalculatedMass	CalculatedMz	Mz	Diff. (mDa)	Diff. (ppm)	DBE
C30 H44 O6	500.3138	501.3211	501.3207	-0.4	-0.7	9.0000

--- End Of Report ---

Figure S29. ^{1}H NMR spectrum of compound **5** (CD_3OD).

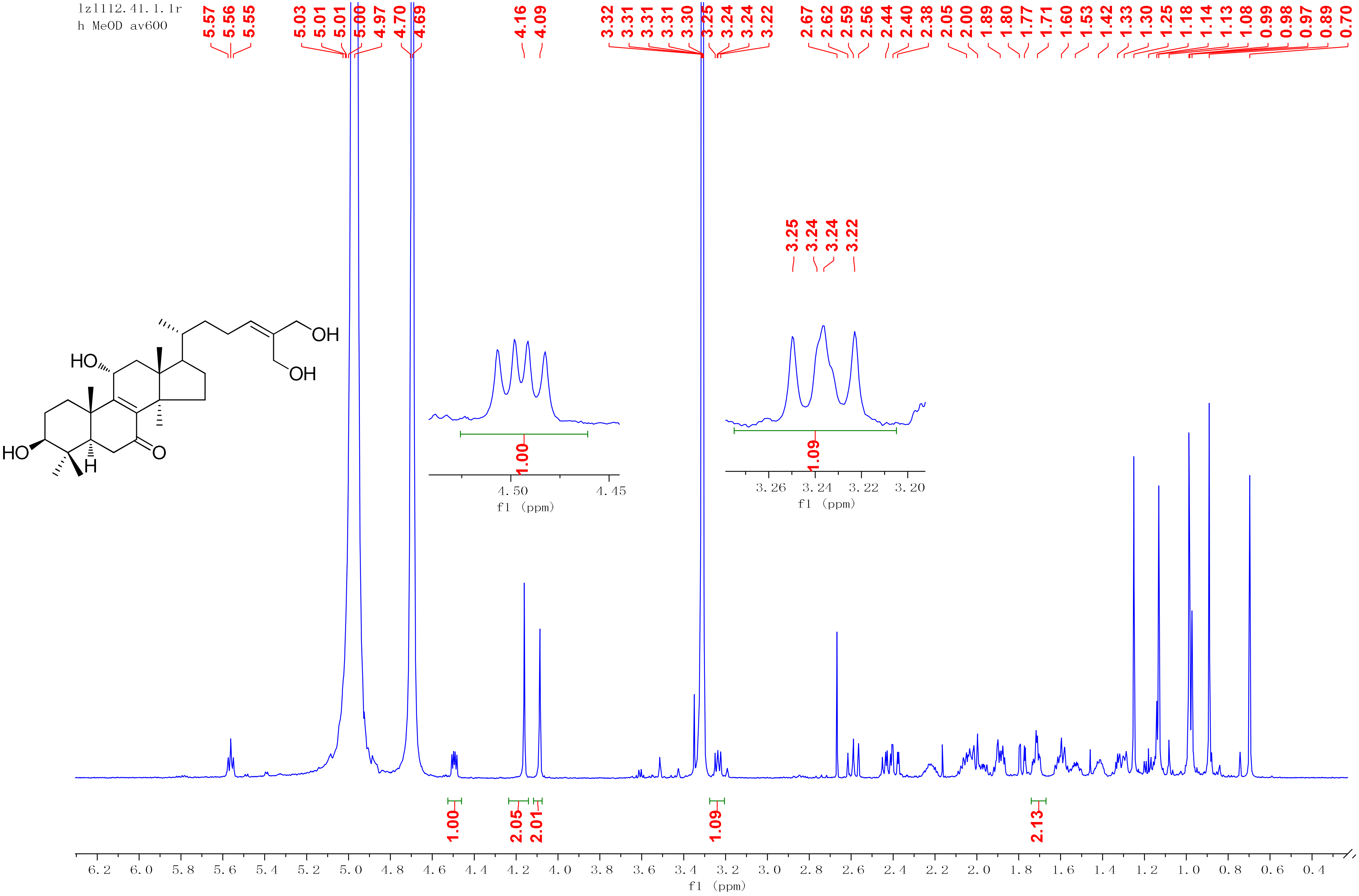

Figure S30. ^{13}C NMR and DEPT spectra of compound **5** (CD_3OD).

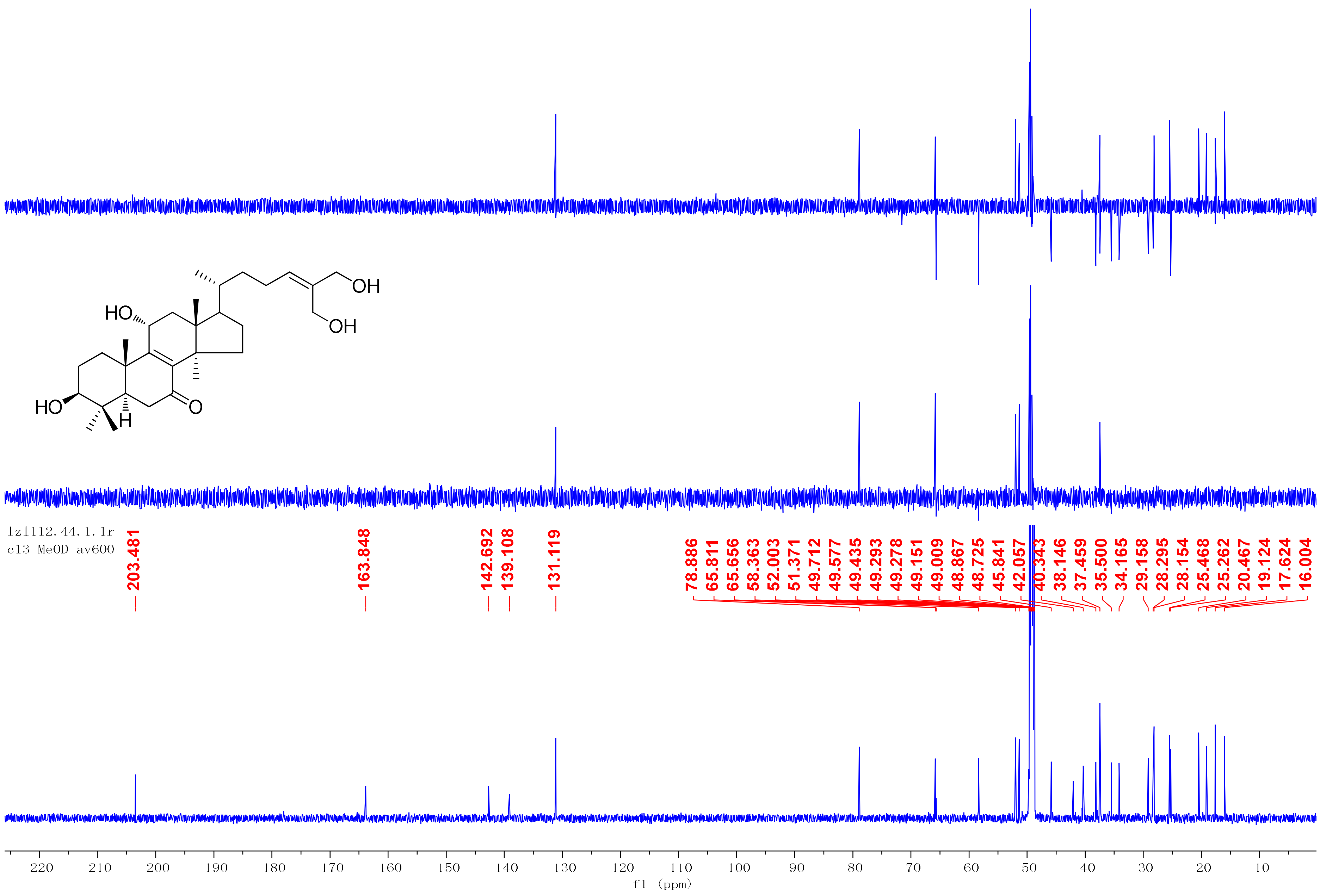

Figure S31. HSQC spectrum of compound **5** (CD_3OD).

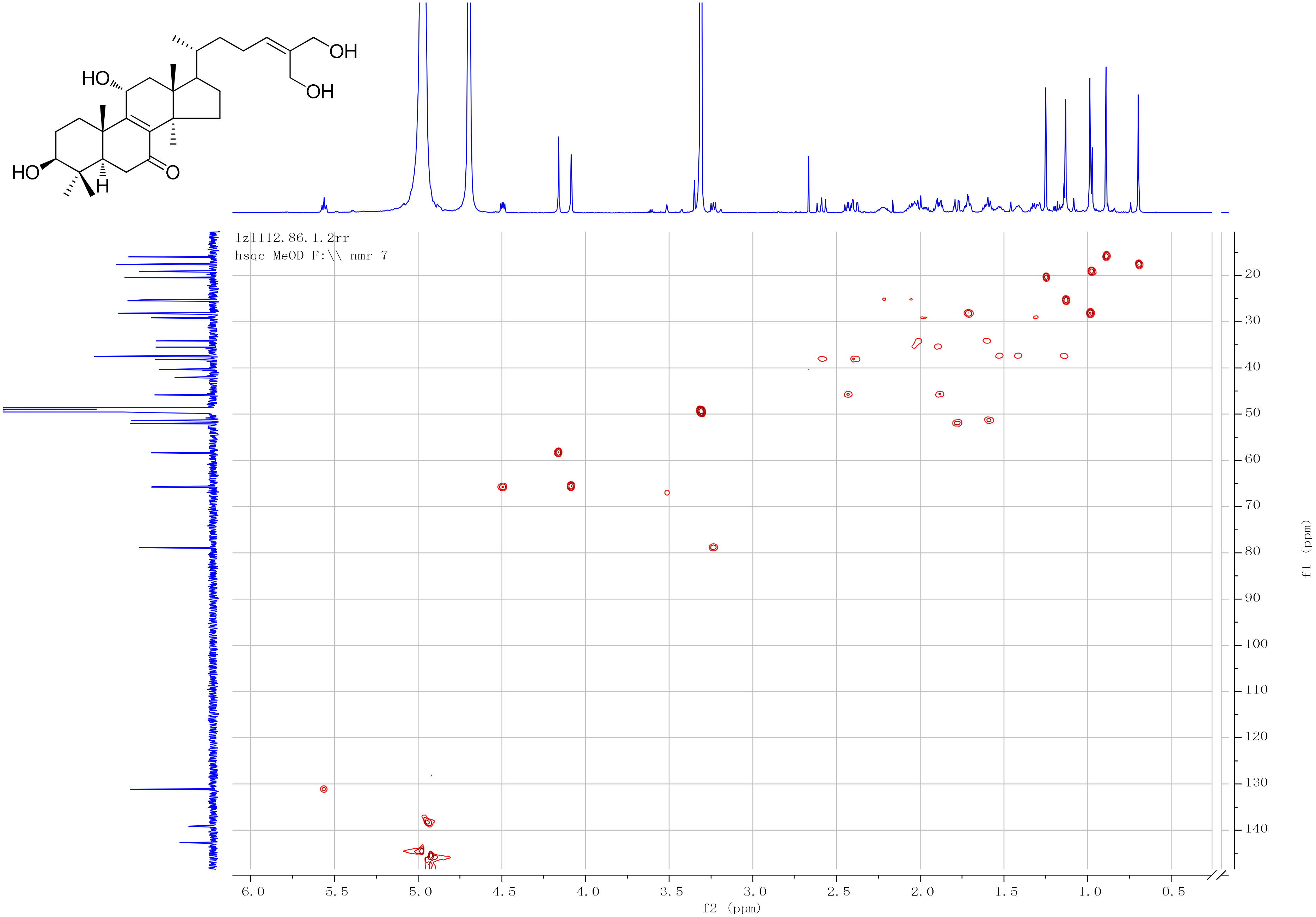

Figure S32. ^{1}H-^{1}H COSY spectrum of compound **5** (CD_3OD).

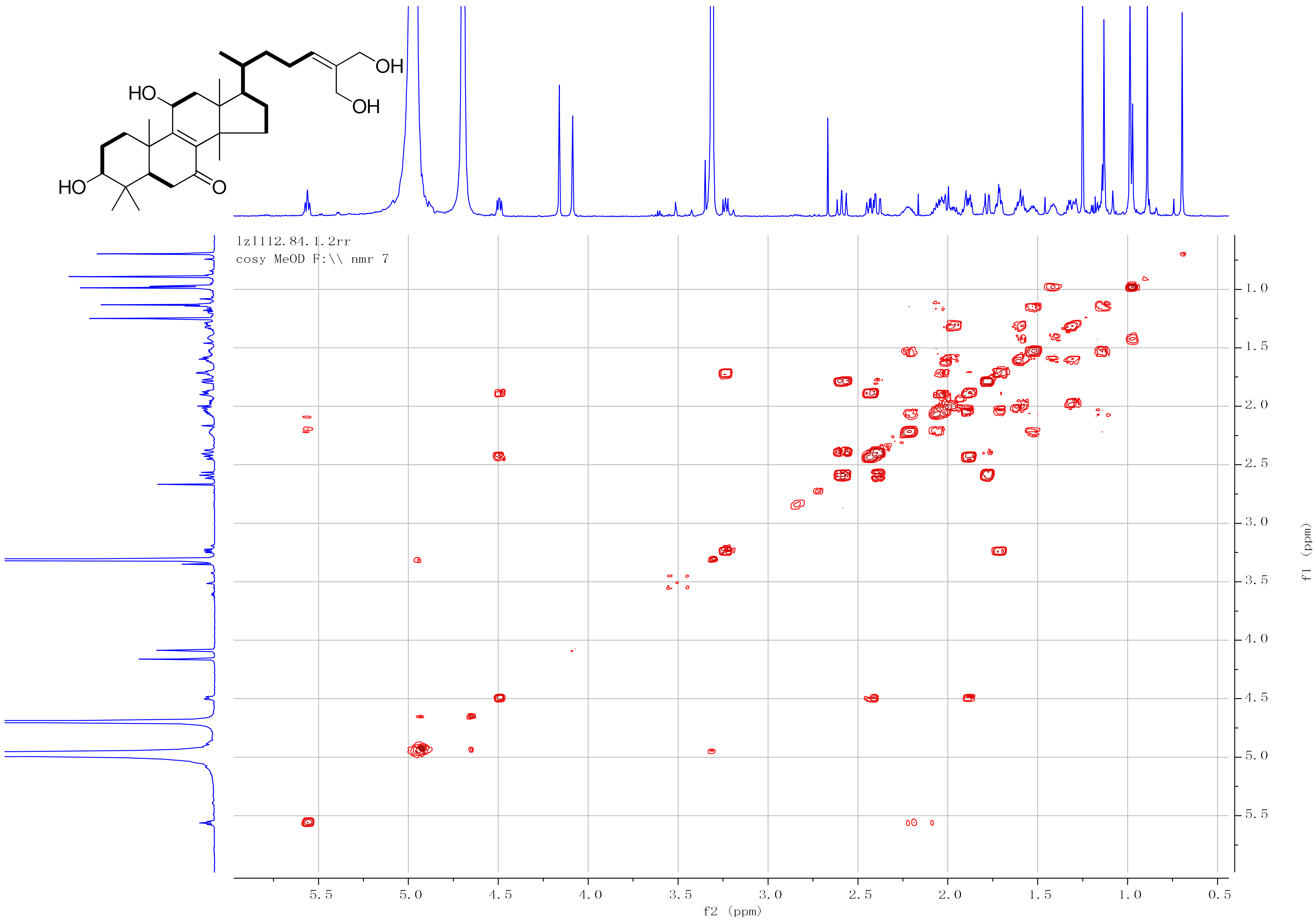

Figure S33. HMBC spectrum of compound **5** (CD_3OD).

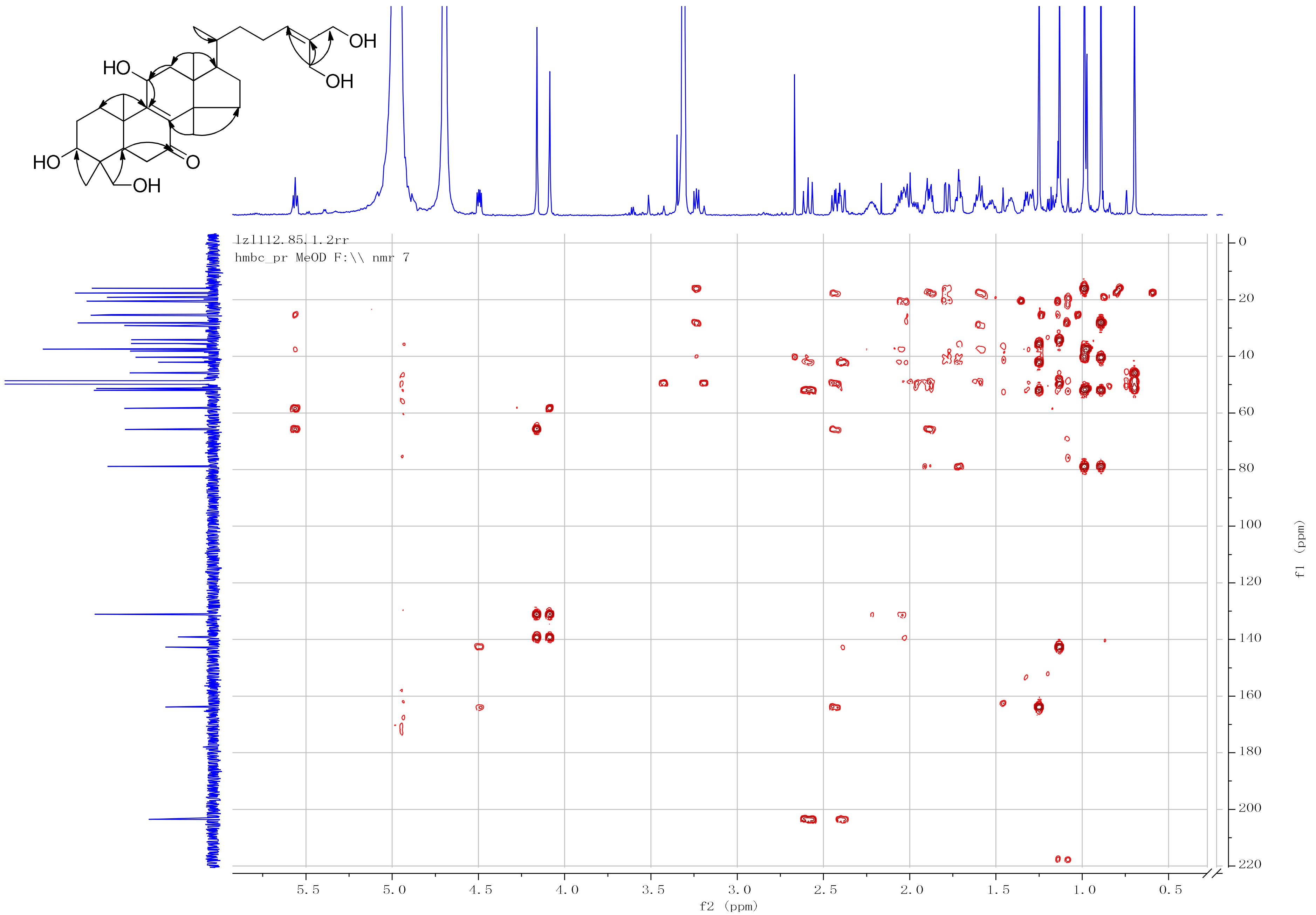

Figure S34. ROESY spectrum of compound **5** (CD_3OD).

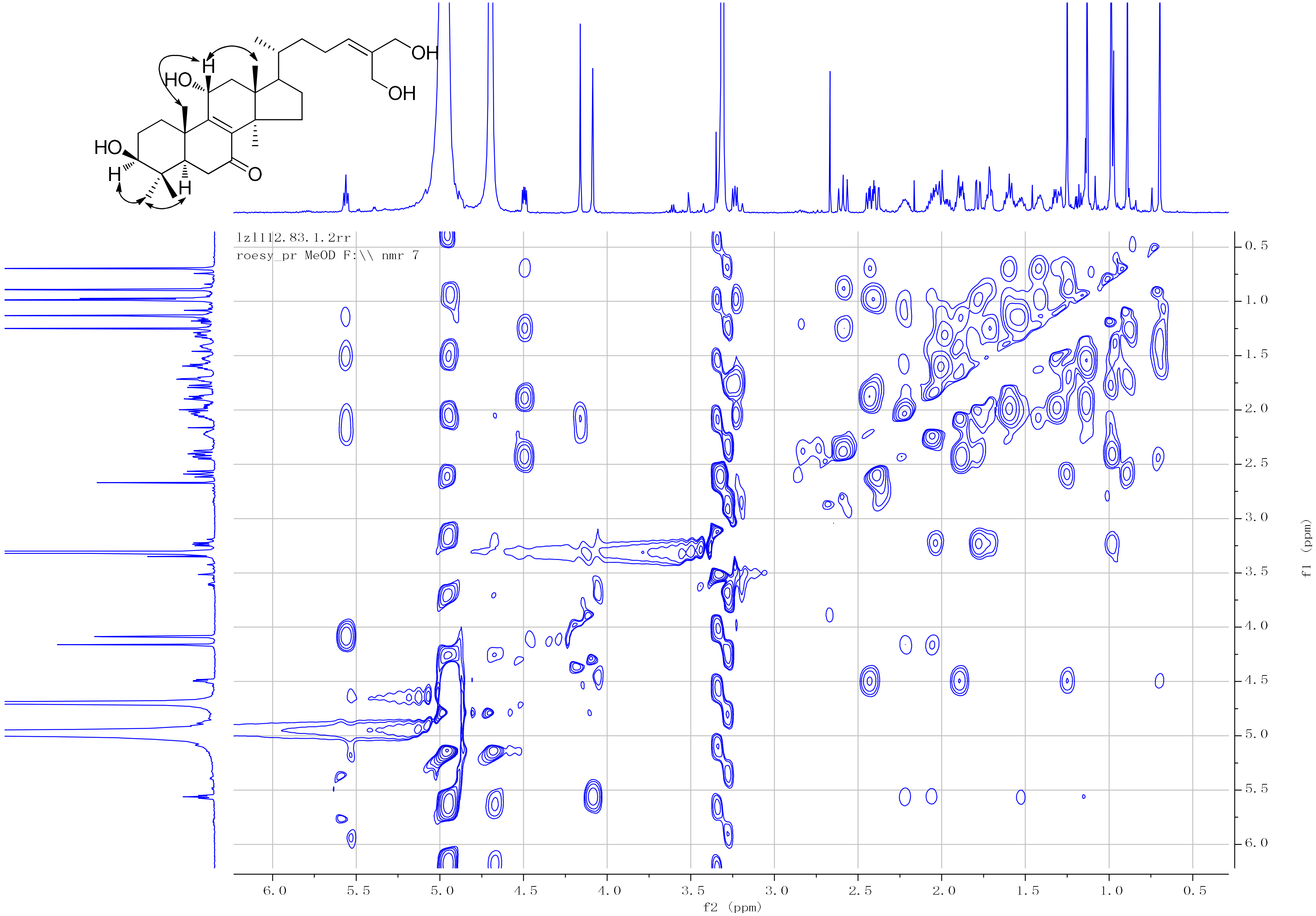

Figure S36. HREIMS spectrum of compound **5**.

Qualitative Analysis Report

**Data Filename**	LZL-112.d	**Sample Name**	LZL-112
**Sample Type**	Sample	**Position**	P1-A9
**Instrument Name**	Instrument 1	**User Name**	
**Acq Method**	SIBU.m	**Acquired Time**	3/30/2015 2:05:09 PM
**IRM Calibration Status**	Success	**DA Method**	Default.m
**Comment**			

**Sample Group**		**Info.**
**Acquisition SW Version**	6200 series TOF/6500 series Q-TOF B.05.01 (B5125.2)	

User Spectra

Fragmentor Voltage	Collision Energy	Ionization Mode
135	0	ESI

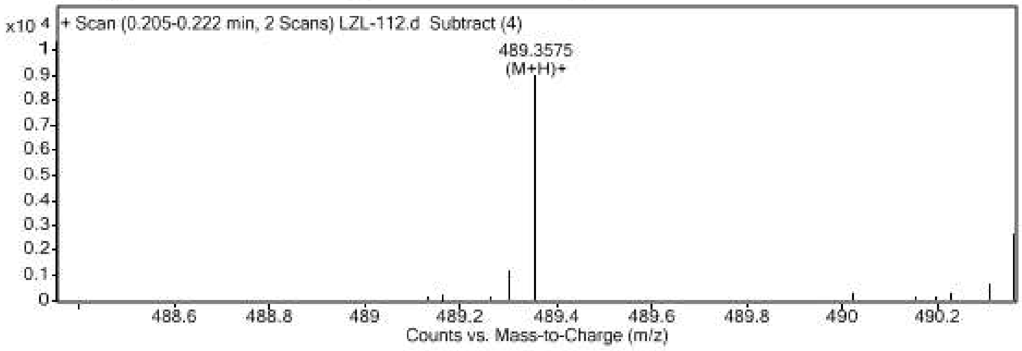

Peak List

m/z	z	Abund
149.0234	1	31267.13
274.2739	1	128177.95
279.1584	1	27487.53
301.1405	1	42847.38
302.3048	1	42036.44
318.3001	1	54158.57

Formula Calculator Element Limits

Element	Min	Max
C	3	60
H	0	120
O	0	30

Formula Calculator Results

Formula	CalculatedMass	CalculatedMz	Mz	Diff. (mDa)	Diff. (ppm)	DBE
C30 H48 O5	488.3502	489.3575	489.3575	0.0	0.1	7.0000

--- End Of Report ---

Figure S36. ^{1}H NMR spectrum of compound **6** (CD_3OD).

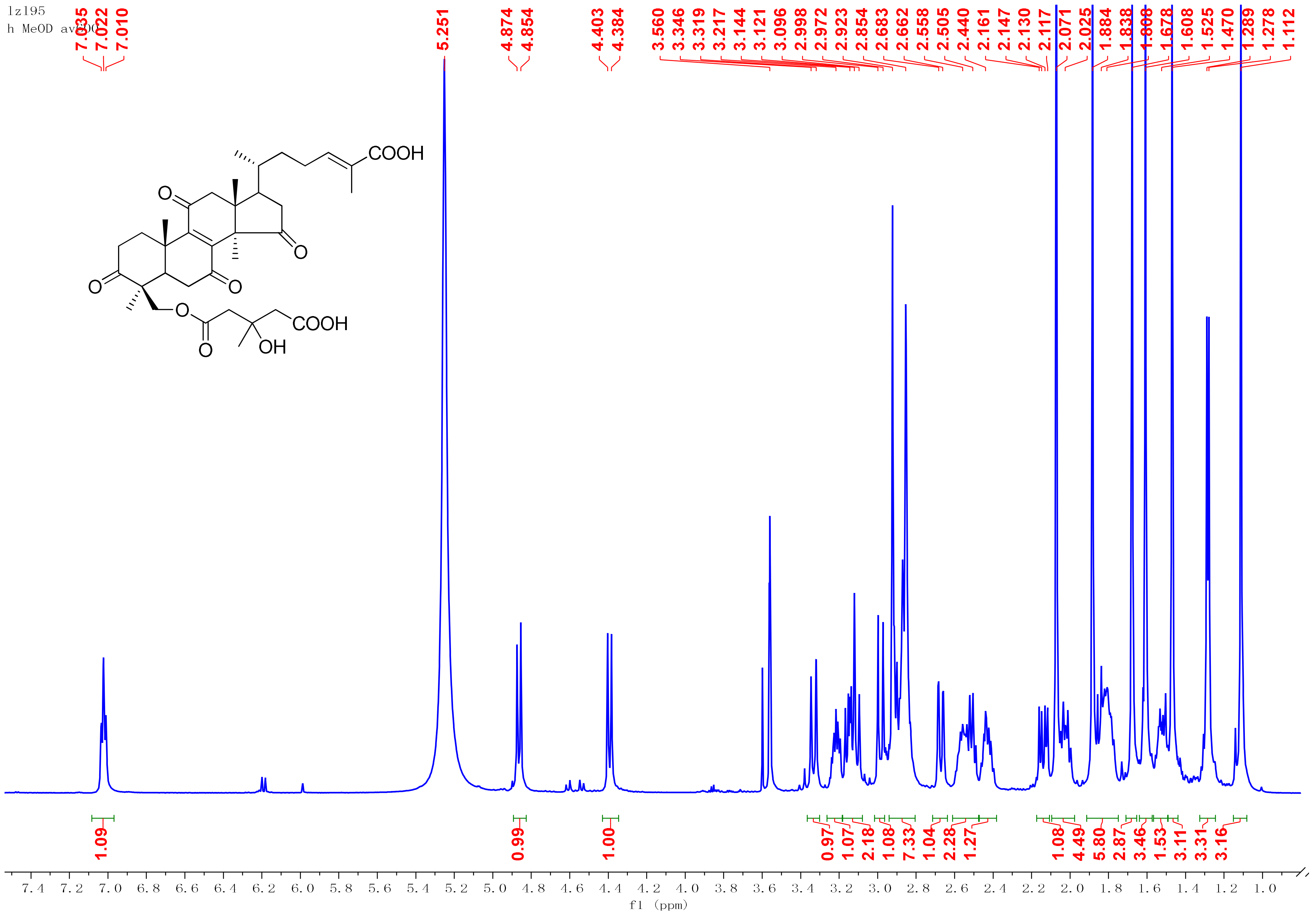

Figure S37. ^{13}C NMR and DEPT spectra of compound **6** (CD_3OD).

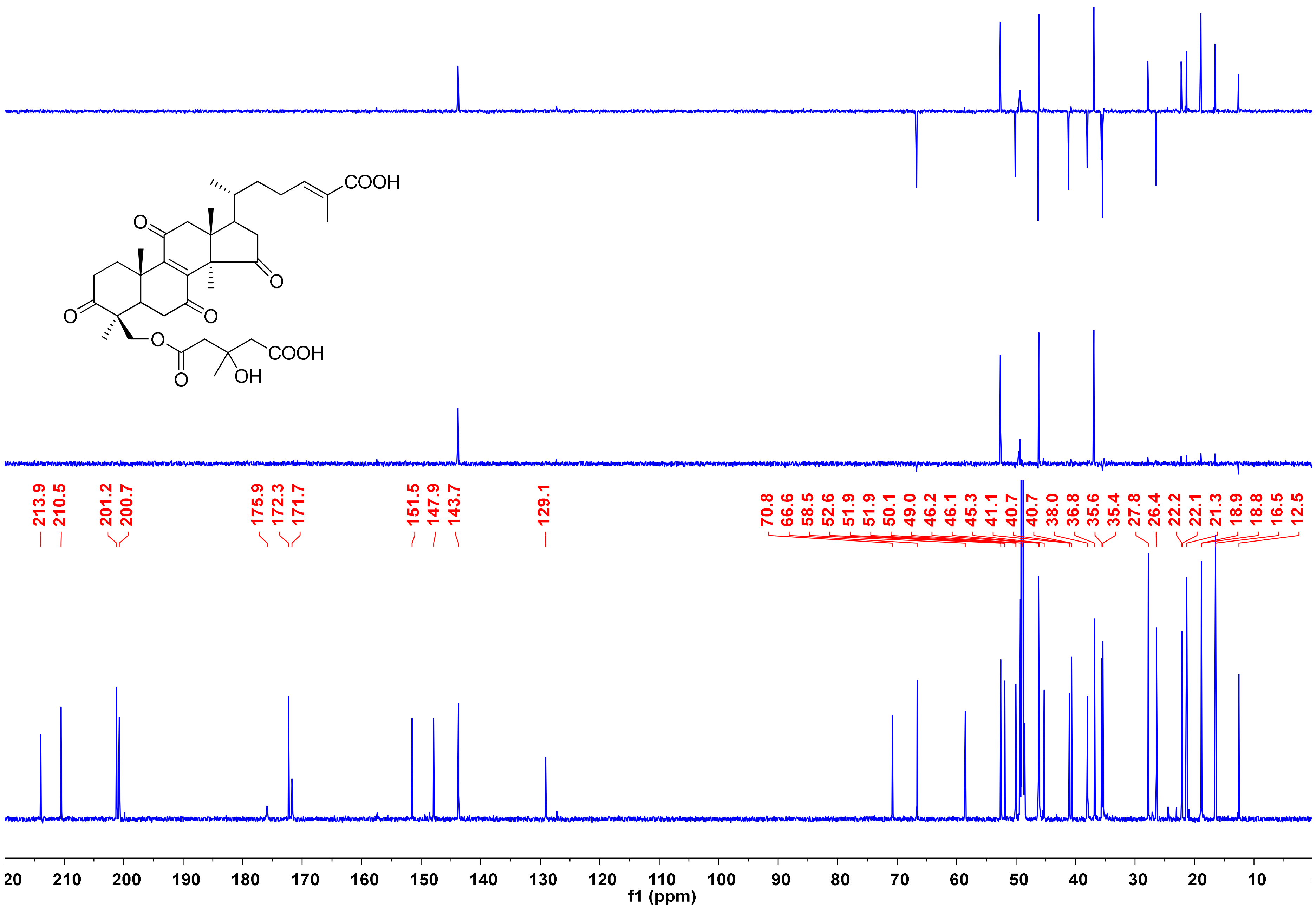

Figure S38. HSQC spectrum of compound **6** (CD_3OD).

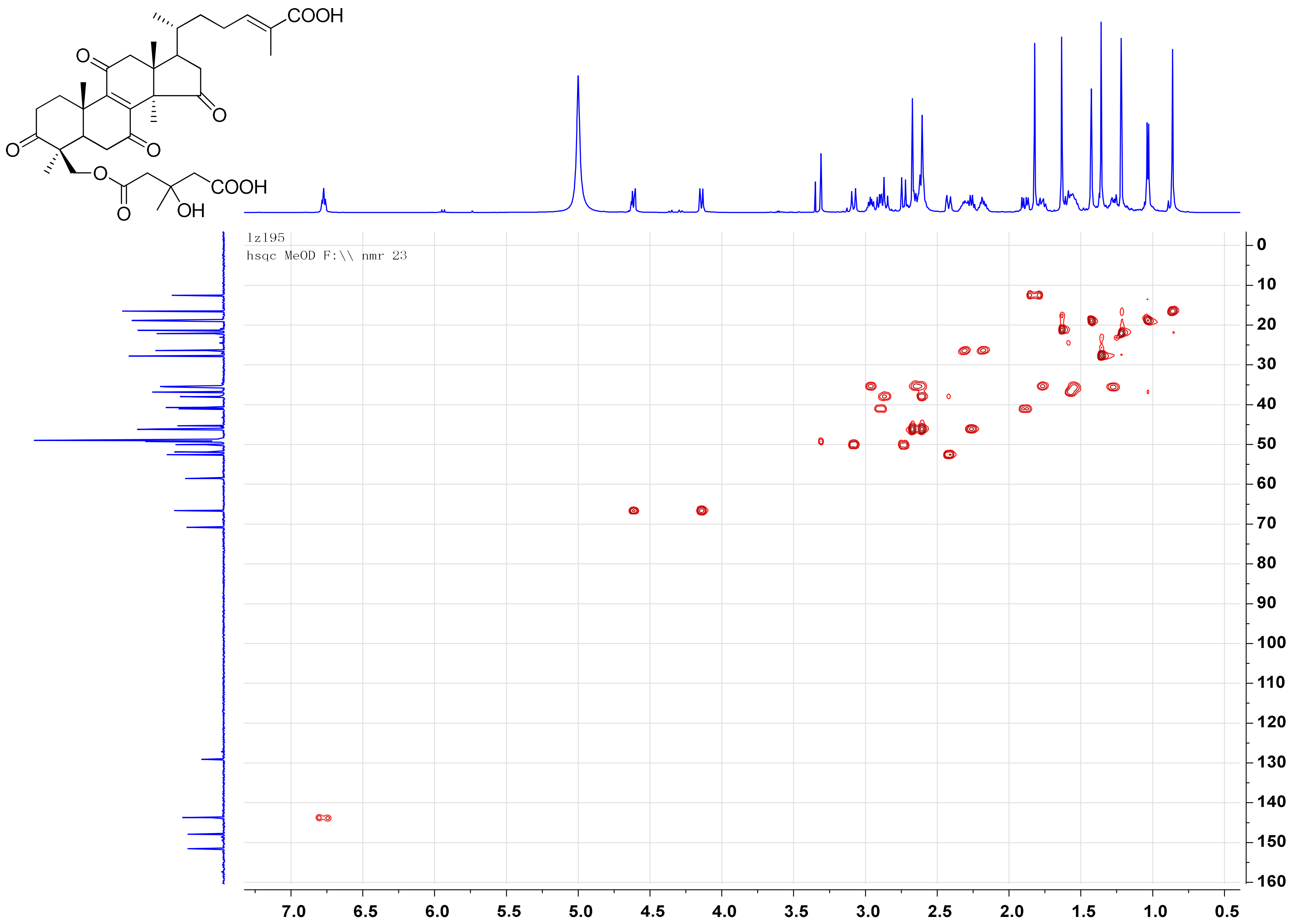

Figure S39. ^{1}H-^{1}H COSY spectrum of compound **18** (CD_3OD).

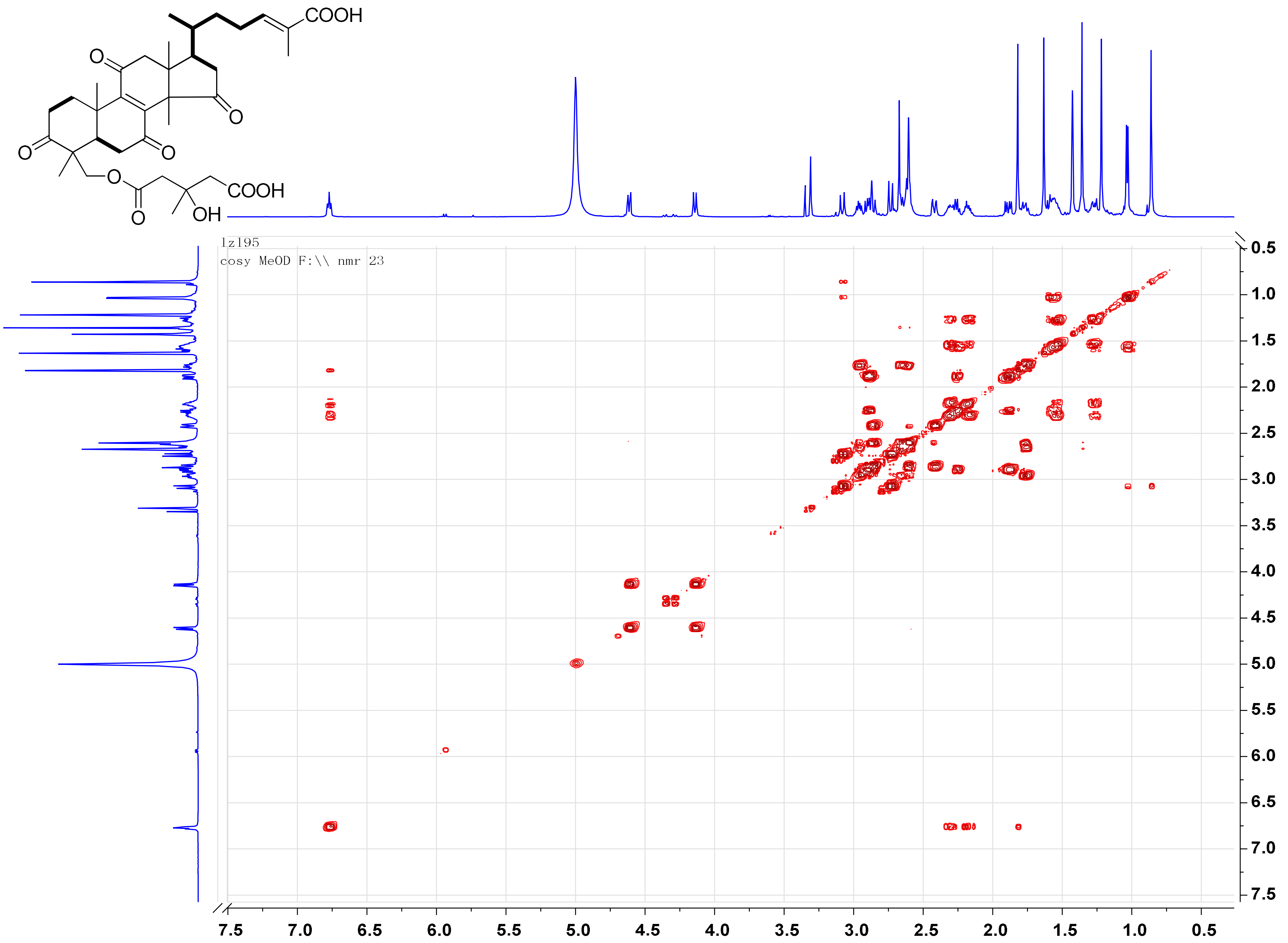

Figure S39. HMBC spectrum of compound **6** (CD_3OD).

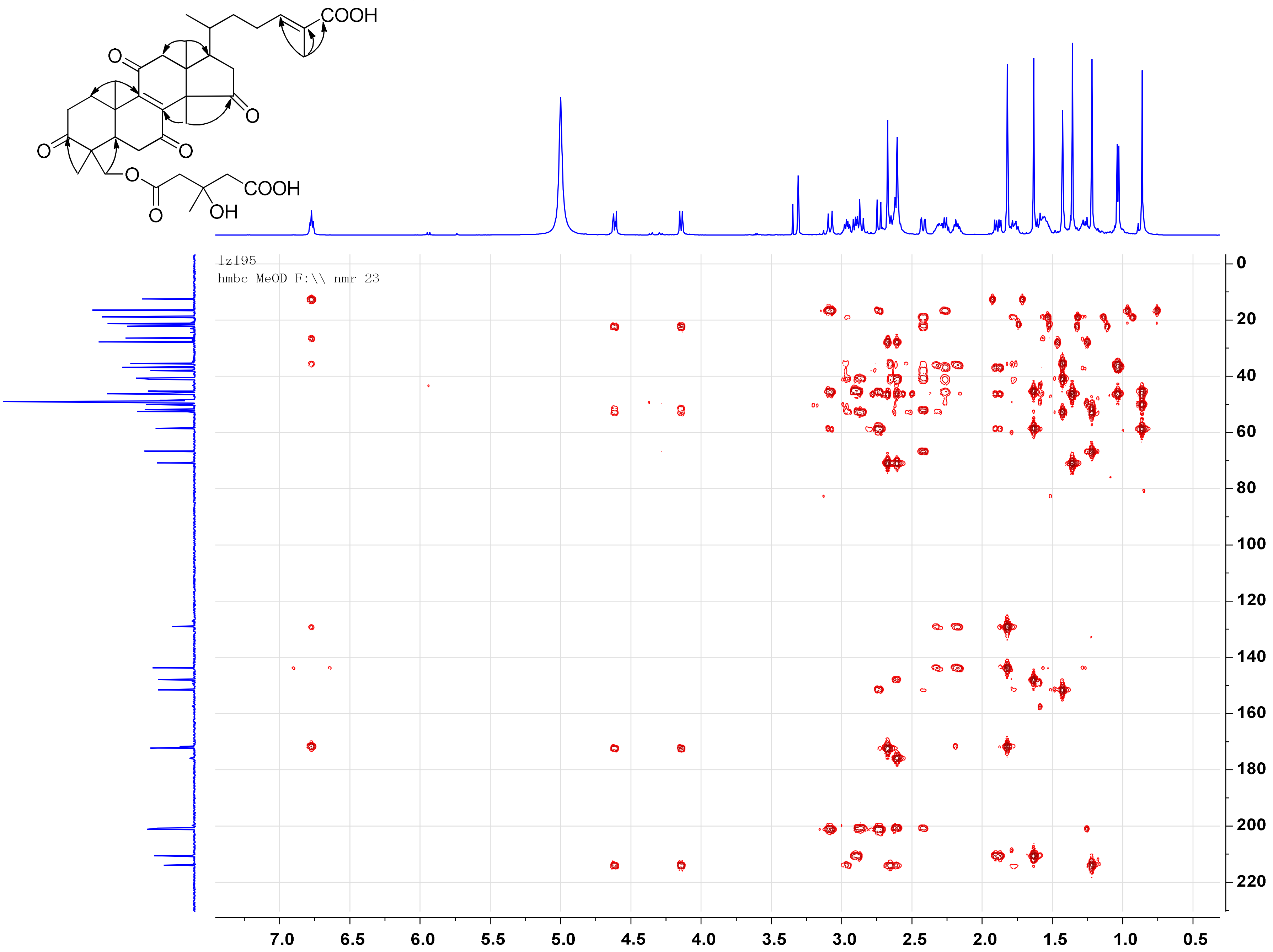

Figure S41. ROESY spectrum of compound **6** (CD_3OD).

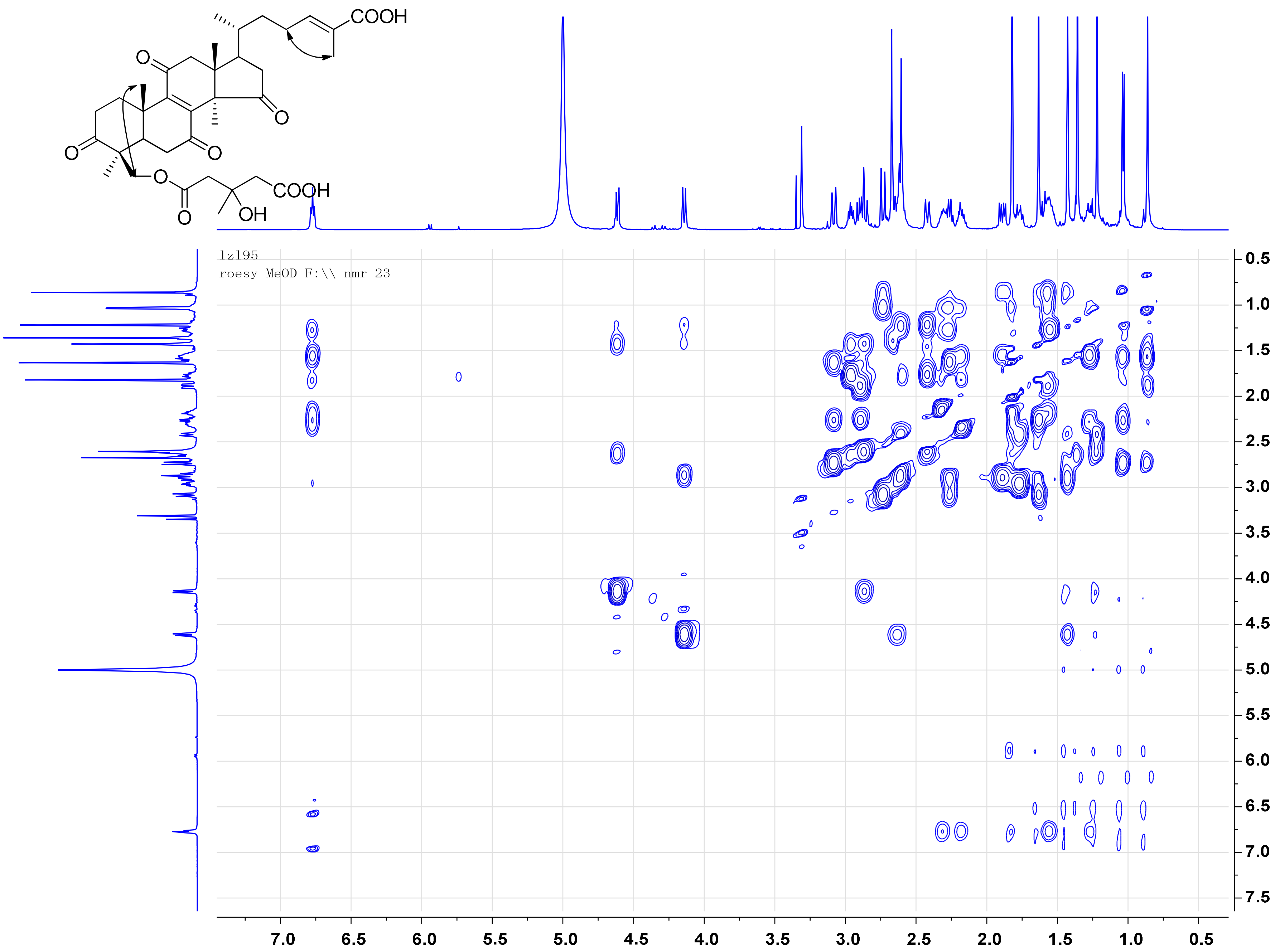

Figure S42. H REIMS spectrum of compound **6**.

Elemental Composition Report

Page 1

Single Mass Analysis

Tolerance = 10.0 PPM / DBE: min = -10.0, max = 120.0
Selected filters: None

Monoisotopic Mass, Odd and Even Electron Ions
23 formula(e) evaluated with 1 results within limits (up to 51 closest results for each mass)
Elements Used:
C: 0-200 H: 0-400 O: 10-12

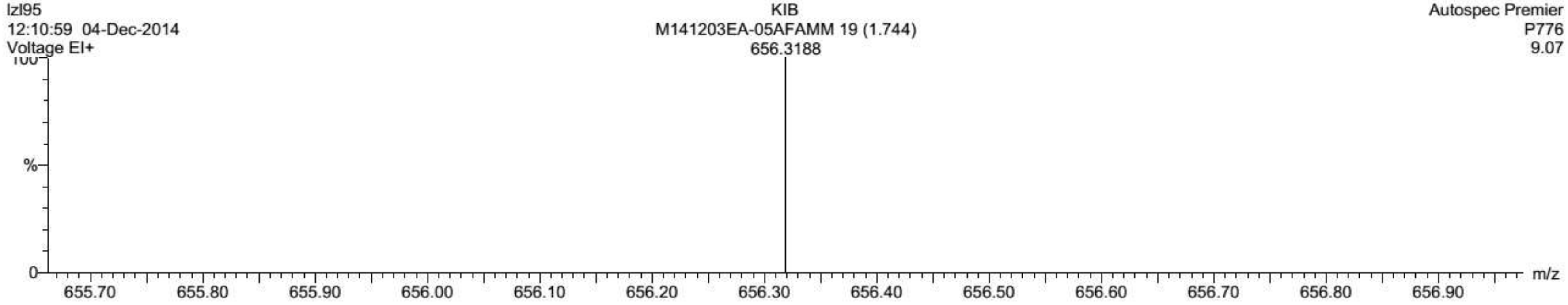

Minimum:				-10.0		
Maximum:		200.0	10.0	120.0		
Mass	Calc. Mass	mDa	PPM	DBE	i-FIT	Formula
656.3188	656.3197	-0.9	-1.4	13.0	5546026.5	C36 H48 O11

Figure S43. ^{13}C NMR spectra of 6 and ganoleucoin L.

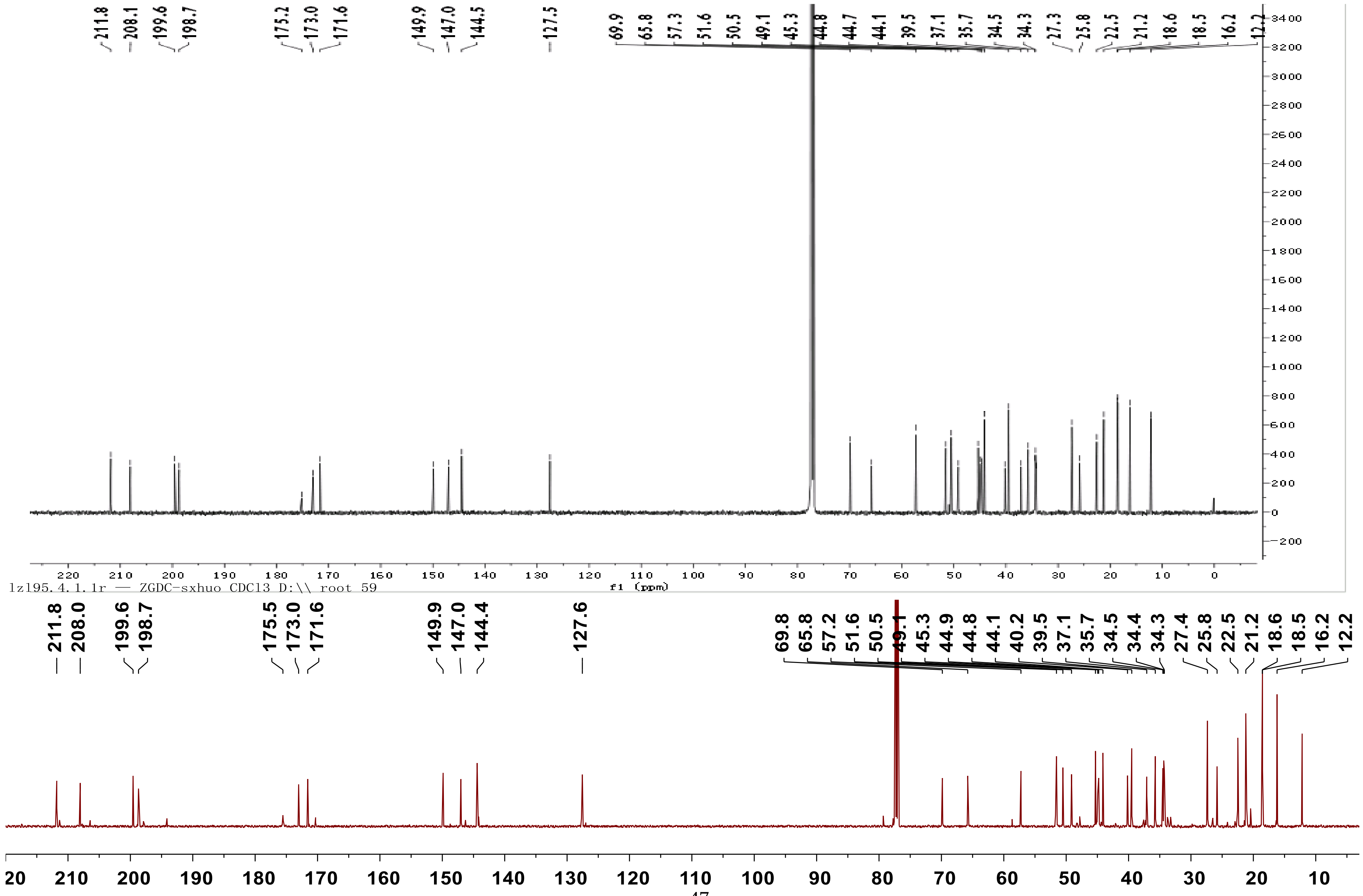

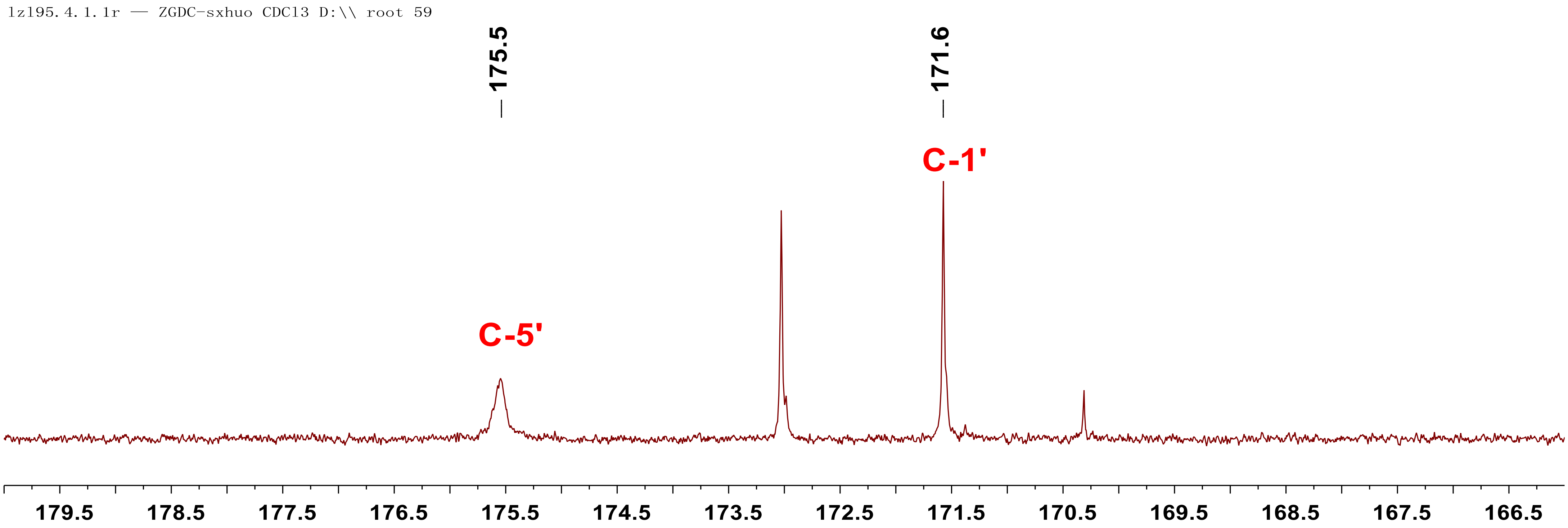
lz195.4.1.1r — ZGDC-sxhuo CDCl3 D:\\ root 59
175.5
171.6
C-1'
C-5'
179.5
178.5
177.5
176.5
175.5
174.5
173.5
172.5
171.5
170.5
169.5
168.5
167.5
166.5

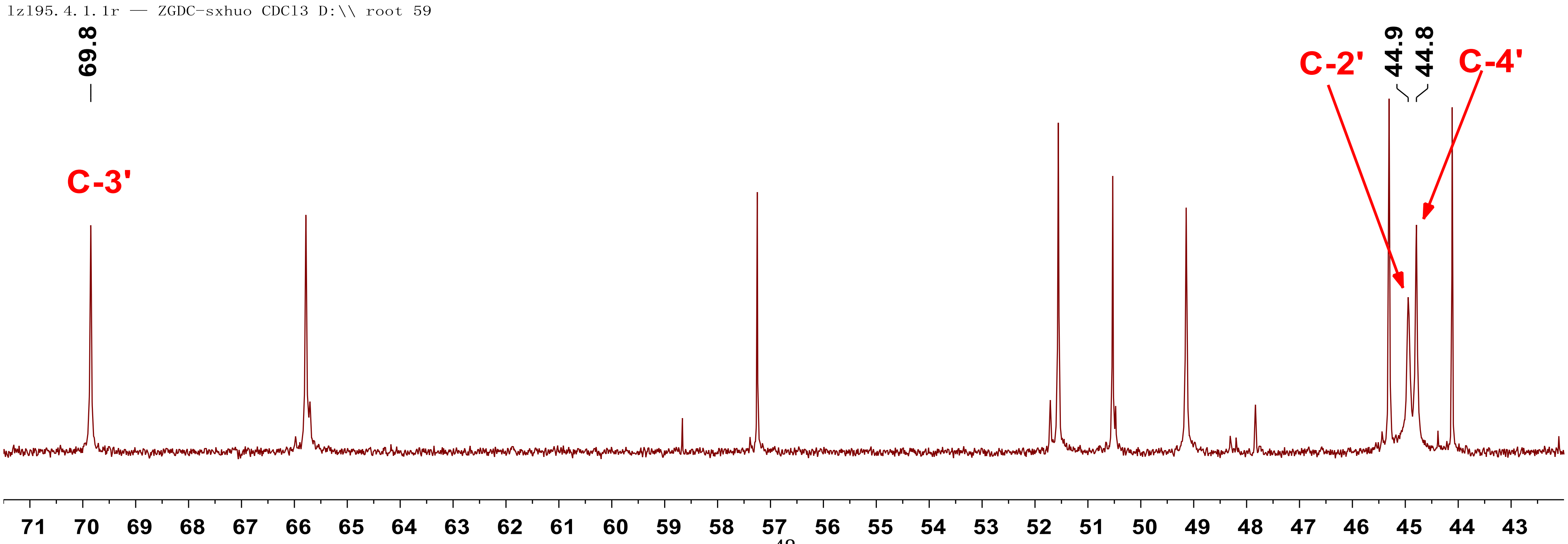
lz195.4.1.1r — ZGDC-sxhuo CDCl3 D:\\ root 59
69.8
44.9
44.8
C-2'
C-4'
C-3'
71
70
69
68
67
66
65
64
63
62
61
60
59
58
57
56
55
54
53
52
51
50
49
48
47
46
45
44
43
